# Transparent PDMS Surfaces with Covalently Attached Lubricants for Enhanced Anti-adhesion Performance

**DOI:** 10.1021/acsami.3c17110

**Published:** 2024-02-13

**Authors:** Tanja Eder, Andreas Mautner, Yufeng Xu, Michael R. Reithofer, Alexander Bismarck, Jia Min Chin

**Affiliations:** † Department of Functional Materials and Catalysis, University of Vienna, Währinger Straße 42, 1090 Vienna, Austria; ‡ Institute of Materials Chemistry and Research, University of Vienna, Währinger Straße 42, 1090 Vienna, Austria; § Institute of Environmental Biotechnology, University of Natural Resources and Life Sciences (BOKU), Konrad-Lorenz-Straße 20, 3430 Tulln, Donau, Austria; ∥ Institute of Inorganic Chemistry, University of Vienna, Währinger Straße 42, 1090 Vienna, Austria; ⊥ Department of Chemical Engineering, Imperial College London, South Kensington Campus, London SW7 2AZ, U.K.

**Keywords:** nonstick coatings, anti-icing, polymer interfaces, liquid repellence, polymer brushes, transparent surfaces

## Abstract

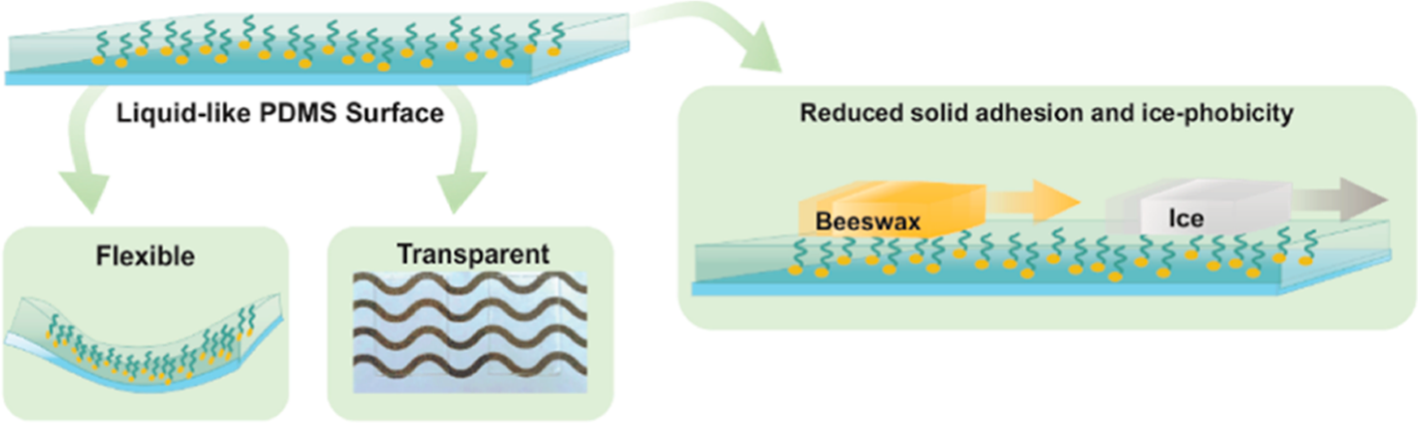

Liquid-like surfaces featuring slippery, omniphobic, covalently attached liquids (SOCALs) reduce unwanted adhesion by providing a molecularly smooth and slippery surface arising from the high mobility of the liquid chains. Such SOCALs are commonly prepared on hard substrates, such as glass, wafers, or metal oxides, despite the importance of nonpolar elastomeric substrates, such as polydimethylsiloxane (**PDMS**) in anti-fouling or nonstick applications. Compared to polar elastomers, hydrophobic **PDMS** elastomer activation and covalent functionalization are significantly more challenging, as **PDMS** tends to display fast hydrophobic recovery upon activation as well as superficial cracking. Through the extraction of excess **PDMS** oligomers and fine-tuning of plasma activation parameters, homogeneously functionalized **PDMS** with fluorinated polysiloxane brushes could be obtained while at the same time reducing crack formation. Polymer brush mobility was increased through the addition of a smaller molecular silane linker to exhibit enhanced dewetting properties and reduced substrate swelling compared to functionalizations featuring hydrocarbon functionalities. Linear polymer brushes were verified by thermogravimetric analysis. The optical properties of **PDMS** remained unaffected by the activation in high-frequency plasma but were impacted by low-frequency plasma. Drastic decreases in solid adhesion of not just complex contaminants but even ice could be shown in horizontal push tests, demonstrating the potential of SOCAL-functionalized **PDMS** surfaces for improved nonstick applications.

## 1 Introduction

High flexibility and stretchability, strong insulating properties, and chemical robustness render elastomeric materials interesting for a wide spectrum of applications ranging from molding applications,^1^ to flow^2,3^ and biomedical devices.^4^ Among these materials, polydimethylsiloxane (**PDMS**) stands out for its heat resistance, exceptional optical transparency, and rapid fabrication.^5^ Surface-exposed methyl groups on the siloxane backbone cause **PDMS** to be inherently hydrophobic and nonpolar. This is crucial for certain uses as it increases biocompatibility in medical devices^6^ and contributes to **PDMS**′ overall chemical inertness.^7^ However, this comes with the drawback of increased solubility for organic solvents or small lipophilic molecules^8^ and swelling upon contact.^9^ Also, the flow properties of aqueous solutions in microfluidics are impeded,^10^ and surfaces experience a higher chance of biofouling.^11^ There is therefore a need to customize **PDMS**′ surface properties depending on its intended application.

**Figure 1:**
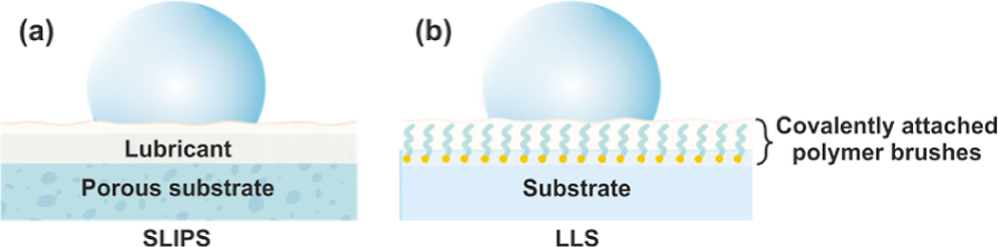
Different strategies to achieve a liquid interface on a solid material. (a) Lubricant wetting of a porous substrate to achieve slippery liquid-infused porous surfaces (SLIPS) and (b) covalent surface functionalization for interfacial liquid behavior.

Recent research suggests that integrating nanoscale liquid behavior to solid surfaces is essential to enhance liquid sliding,^12,13^ minimize adhesion of contaminants,^14−16^ reduce biofouling,^17^ or improve intermembrane transport.^18^ Conventionally, achieving a liquid interface on **PDMS** has been done by swelling of the **PDMS** network with lubricants,^19^ thus creating SLIPS (Figure 1a). While SLIPS can reestablish their surface and heal once disturbed as the fluid flows back after displacement^14,20^ and are virtually defect-free, they are also prone to lubricant depletion and thus have limited durability, as their lubricant retention relies on physical rather than covalent interactions.^21,22^ Another approach is to incorporate hemitelechelic polymers, which have a reactive end group, into the bulk polymer matrix to endow lubricating properties, but this requires laborious coating or gel engineering of formulations to obtain the desired mechanical properties.^23^ Further, this results in the modification of the entire bulk of the material, rather than in engineering the material’s surface properties. An ideal approach would be to develop a method suitable for equipping prefabricated elastomers with liquid interfaces to expand their potential range of applications.

One route for this would be covalent attachment of lubricating molecules such as linear **PDMS** to the substrate surface^24^ (Figure 1b). Compared to carbon-based polymers, the siloxane backbone offers great rotational flexibility^18,25^ as well as low glass transition temperatures, thus tending toward liquid-like behavior at room temperature.^25,26^ Current research has focused predominantly on hard and polar substrates,^27^ such as silicon wafers,^12,13,16,22,26^ glass,^13,16,22^ aluminum,^22^ or stainless steel substrates,^18^ as they offer uniform and smooth surfaces, avoiding liquid pinning problems. Their already oxygen-rich surfaces additionally facilitate chemical functionalization. Two key methods are usually employed to achieve liquid-like surfaces (LLS) on nonporous hard substrates: the first route involves the grafting-to of polymers that bear one reactive chain end group, typically monofunctionally terminated silicone oil^28,29^ or even unreactive silicone oil.^25^ However, the chain lengths of the grafted polymers inherently limit the resulting liquid film thickness, and their steric hindrance can obscure potential grafting sites, thereby reducing surface coverage.^30,31^ In the second route, a grafting-from case, smaller molecular precursors, usually various silane precursors, are used to grow longer chains on the substrate,^29^ allowing for higher grafting densities by avoiding diffusion obstruction.^13,16,29,30^ A salient consideration is the type of silane precursor used—silanization of PDMS typically involves silanes bearing either one or three hydrolyzable groups. This results in the grafting of a silane monolayer or the generation of a cross-linked, immobile siloxane multilayer,^32,33^ both of which limit LLS formation. To avoid cross-linking, achieve linear polymerization, and maximize subsequent interfacial slip, silanes with two hydrolyzable groups are instead required.^32,34^ The remaining two organic functional groups can then bear different moieties, so tunable functionalities can be integrated into the polymer brushes based on the precursor used, which we demonstrate in this work.

**Figure 2:**
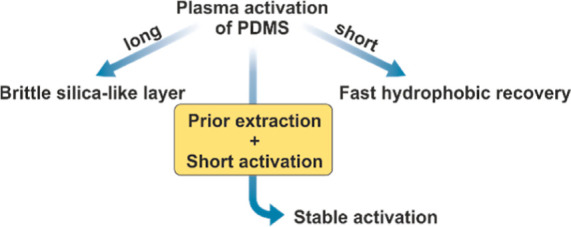
Challenges in **PDMS** plasma activation.

However, adapting these concepts to soft substrates such as **PDMS** requires careful consideration. Hydrophobic **PDMS** requires activation prior to chemical functionalization.^35^ Plasma or UV/ozone introduces the necessary functional groups, such as hydroxyl groups, accessible for chemical binding, but the oxidizing environment frequently leads to the formation of a hard silica-like layer on soft **PDMS** substrates.^36^ Activated **PDMS** is susceptible to surface cracking, especially during mechanical deformation due to the mismatch of mechanical properties between soft **PDMS** relative to the generated silica-like surface.^37^ This leads to a prominent challenge known as “hydrophobic recovery” as **PDMS** reverts its surface chemistry due to the diffusion of low molecular weight (LMW) species from the bulk through the cracks to the surface.^38^ Additionally, cracks in the silica-like layer increase inhomogeneity of the surface, and optical properties may suffer from the presence of cracks as light transmission is altered between the two layers causing light scattering.^37^ These prominent issues can be addressed by shorter plasma activation times to minimize the formation of silica-like layers but comes at the cost of either incomplete surface activation or faster hydrophobic recovery, as high-mobility **PDMS** chains can quickly reorient or redistribute to minimize surface energy^39,40^ (Figure 2).

This reported work aims to explore the adaptation of LLS from hard substrates to elastomeric **PDMS** substrates. We target a reduction in the formation of the silica layer and hydrophobic recovery through a preceding extraction step of the LMW species combined with careful control over the **PDMS** plasma activation parameters. These steps enable straightforward covalent grafting of liquid-like linear polysiloxane brushes via the grafting-from method by dip-coating. Different moieties of the brushes are provided by the employed silanes. This approach equips commercially available silicone elastomer substrates with increased droplet mobility, anti-adhesion, and anti-icing properties.

## 2 Results and Discussion

### 2.1 Substrate Preparation and Functionalization

**Figure 3:**
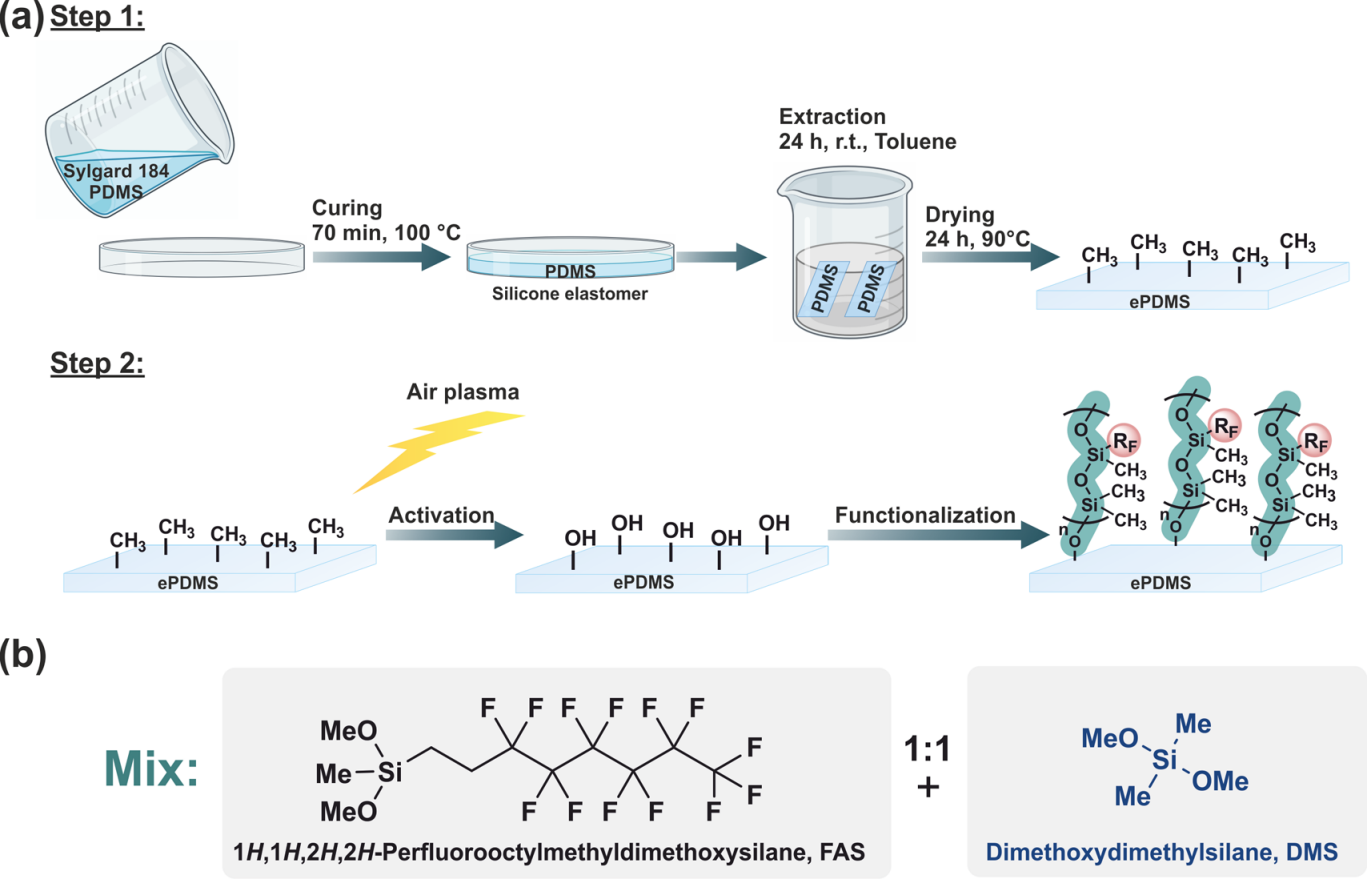
(a) Scheme of the sample preparation process. Step 1 depicts the preparation of **PDMS** elastomer sheets and the subsequent extraction process in toluene. In step 2, samples are activated with air plasma and dip-coated to graft polymer brushes to the surface. (b) Silanes and abbreviations utilized in **PDMS** functionalization.

**PDMS** samples were prepared (Figure 3a) by using a commercially available **PDMS** prepolymer mixture (Sylgard 184) through a hydrosilylation reaction with a platinum catalyst. Besides the main framework components, commercial mixtures also include additives, such as fillers, cross-linking inhibitors, and solvents,^5^ which contribute to the presence of mobile LMW species and uncross-linked oligomers within the final elastomer.^9,41^ LMW species were extracted by immersion in toluene from all silicone elastomer substrates for 24 h and are subsequently denoted as “**ePDMS**” (Section 4.2.1, Table S2, Figure S2).

To investigate the effect of plasma activation parameters on the formation of silica-like layers, the elastomer samples were activated in air (0.14 mbar) for an exposure time of 60 s at either 75, 150, or 225 W for 13.56 MHz (HF) plasma and 50, 100, or 150 W for 40 kHz (LF) plasma. After plasma treatment, the **PDMS** and **ePDMS** surfaces became hydrophilic, allowing for complete wetting by water. Removal of LMW fragments by extraction extended the longevity of **PDMS** activation by retarding hydrophobic recovery,^42^ as water droplets on the surface still possessed a low contact angle 20 h after plasma exposure (Figure S3).

After activation, the **ePDMS** was functionalized, adapting a published procedure^16^ by initially dip-coating an acidified silane in isopropanol solution and polymerizing of silanes upon concentration during drying. The silanes utilized were fluoroalkylsilane 1*H*,1*H*,2*H*,2*H*-perfluorooctylmethyldimethoxysilane (**FAS**) and dimethoxydimethylsilane (**DMS**). Additionally, a coating mixture, in which the silane portion of the solution comprising 50 wt % **FAS** and 50 wt % **DMS** to serve as a smaller linker molecule in between bulkier **FAS**, potentially allowing longer siloxane chain formation, was investigated. This functionalization is denoted as **Mix** (Figure 3b). Correspondingly, the functionalized **ePDMS** substrates are denoted with the employed silane as subscript: **ePDMS**_**FAS**_, **ePDMS**_**DMS**_, and **ePDMS**_**Mix**_. The dipped specimens were air-dried for 20 min, followed by rinsing to remove unreacted silane (Section 4.2.2). The solvent readily successfully dewetted functionalized surfaces during washing, unlike for unfunctionalized **ePDMS**. As a reference, regular (unextracted) **PDMS** also underwent functionalization with **FAS** and is referred to as **PDMS**_**FAS**_.

### 2.2 Wetting Properties and Surface Tension of Functionalized Substrates

As previously demonstrated in slippery omniphobic covalently attached liquid coatings on glass substrates,^16^ bifunctional silanes should yield linear siloxane chain growth^34^ and form LLSs with interfacial slip and low friction. Contact angle hysteresis (Δθ = θ_a_ – θ_r_) as the difference between advancing (θ_a_) and receding contact angle (θ_r_) has recently been suggested as the prime indicator for such behavior.^43^ For water, θ_a_ showed little variation between substrates, while significant differences in θ_r_ and thus Δθ were found (Table 1). For the investigated substrates, θ_r_ on **ePDMS** were the lowest, as droplet withdrawal was obstructed by surface features, leading to dewetting at lower angles. θ_r_ increased for **ePDMS**_**DMS**_ as the chain mobility arising from the grafted PDMS chains aided droplet depinning. θ_r_ also increased for **ePDMS**_**FAS**_ as low surface energy groups decreased the work of adhesion between the test liquid and the surface. For **ePDMS**_**Mix**_, we observed further increased θ_r_, which we attributed to a synergistic effect of low surface energy groups and increased chain mobility, as partial **FAS** replacement with less sterically demanding **DMS** increased interfacial slip of the resulting LLS (Figure S4). Dynamic contact angles for diiodomethane can be interpreted in a similar manner, although the variation in θ_a_ was more pronounced due to fluorination of **ePDMS**_**FAS**_ and **ePDMS**_**Mix**_ as well as the higher surface roughness of **ePDMS**. For hexadecane, no dynamic contact angles could be obtained for **ePDMS** or **ePDMS**_**DMS**_ due to the immediate swelling of the substrates by the probing liquid. Swelling of the substrates was prevented in **ePDMS**_**FAS**_ and **ePDMS**_**Mix**_ due to fluorination of the polymer brushes (Figure S5). The lower degree of fluorination in the **Mix** functionalization resulted in a lower θ_a_, while the θ_r_ remained similar. Surface tensions (γ_sv_) were calculated from advancing contact angles (θ_a_), as they are sensitive to the low surface energy part of a surface.^44^ The Owens, Wendt, Rabel, and Kaelble (OWRK) method^45^ was employed for water (γ_lv_ = 72.8 mN/m of which γ_lv_^P^ = 51.0 mN/m and γ_lv_^D^ = 21.8 mN/m) and diiodomethane (γ_lv_ = 50.8 mN/m of which γ_lv_^D^ = 49.0 mN/m and γ_lv_^P^ = 1.8 mN/m^46^) as test liquids (Table 1). For **ePDMS** γ_sv_ = 17.3 mN/m is obtained, which is lower compared to the commonly reported literature values for PDMS of ∼21 mN/m.^5,47^**PDMS** extraction led to increased surface roughness already indicated by increased θ_a_ and Δθ and consequently lower calculated γ_sv_. When comparing functionalized **ePDMS** substrates, calculated γ_sv_ correlates with the increasing degree of fluorination of **ePDMS**_**DMS**_, **ePDMS**_**Mix**_, and **ePDMS**_**FAS**_, respectively. For **ePDMS**_**FAS**_, γ_sv_ = 15.0 mN/m is in good agreement with the γ_sv_ of fluorosilicones (14–15 mN/m^48^).

**Table 1:** Advancing Contact Angles (θ_a_), Receding Contact Angles (θ_r_), Contact Angle Hysteresis (Δθ) for Water, Diiodomethane, and Hexadecane (Hex) and Surface Tension (γ_sv_) Calculated According to OWRK Method^a^

Sample	θ_a,H_2_O_	θ_r,H_2_O_	Δθ_H_2_O_	θ_a,CH_2_I_2__	θ_r,CH_2_I_2__	θ_CH_2_I_2__	θ_a,Hex_	θ_r,Hex_	Δθ_Hex_	γ_sv_ [mN/m]
ePDMS_DMS_	108.0 ± 1.8°	80.0 ± 1.0°	28.0°	66.9 ± 2.0°	50.3 ± 1.1°	16.6°	—	—	—	25.1
ePDMS_FAS_	114.6 ± 1.4°	87.0 ± 1.0°	27.9°	85.1 ± 1.6°	47.8 ± 1.5°	37.3°	77.8 ± 1.3°	34.8 ± 1.6°	43.7°	15.0
ePDMS_Mix_	112.2 ± 1.0°	93.6 ± 2.0°	18.5°	73.4 ± 1.3°	62.6 ± 1.3°	10.8°	57.1 ± 1.4°	33.6 ± 1.8°	23.5°	21.5
ePDMS	113.2 ± 0.6°	75.7 ± 1.6°	37.5°	93.4 ± 0.5°	53.2 ± 0.4°	40.2°	—	—	—	17.3

a Dynamic contact angles of hexadecane could not be measured on **ePDMS** and **ePDMS**_**DMS**_ due to substrate swelling, indicated by “—”

**Figure 4:**
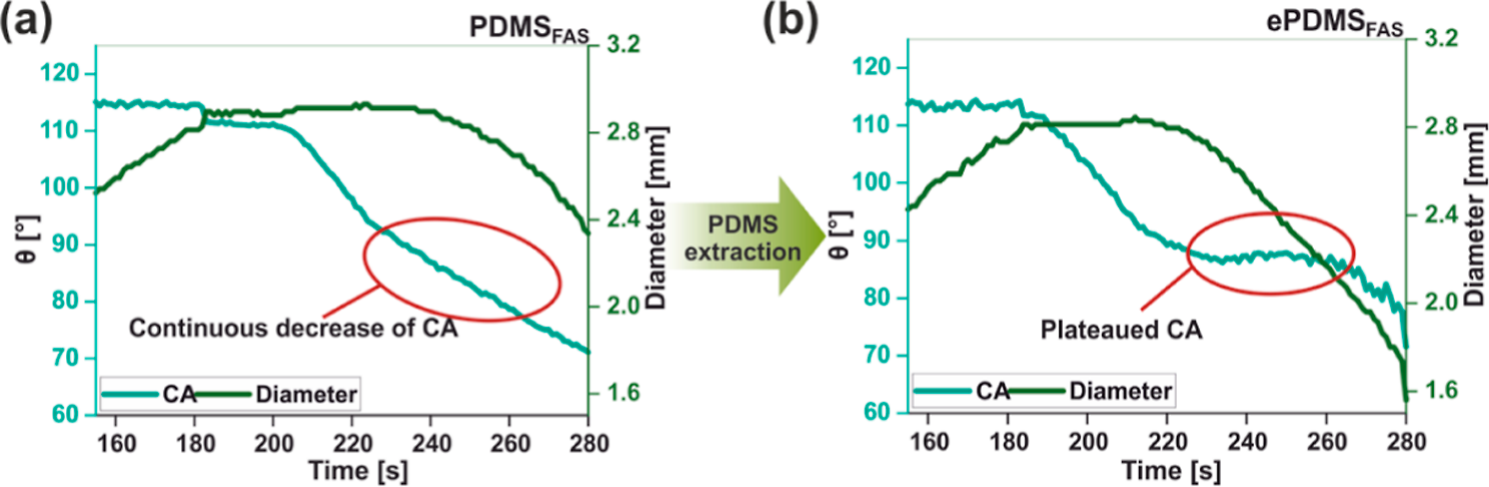
Results of contact angle measurements. (a) Contact angle (θ) and droplet diameter during dynamic water contact angle measurements on **PDMS**_**FAS**_, θ continuously decreases in the circled region; (b) upon extraction, a stable (plateaued) θ_r_ is apparent on **ePDMS**_**FAS**_.

Δθ further serves as a measure for surface heterogeneity; Δθ typically increases for heterogeneous surfaces due to pinning and depinning of the contact line on contrasting surface chemistries.^49^ Dynamic contact angle measurements were carried out to compare the surface homogeneities of **PDMS**_**FAS**_ and **ePDMS**_**FAS**_. For **PDMS**_**FAS**_, no θ_r_ was measurable due to droplet contact line pinning during probing liquid aspiration, as indicated in Figure 4a. Impaired dewetting on functionalized **PDMS** is caused by uneven functionalization^44^ as unextracted and more mobile LMW chains contribute to hydrophobic recovery but also react with and deplete coating reagents. For **ePDMS**_**FAS**_, a stable θ_r_ value was observed for more than 30 s during dewetting (Figure 4b), indicating that the extraction step produced a homogeneously functionalized surface.

For the different substrates, sliding angles (α) follow the same trend as Δθ, apart from that of hexadecane (Table 2). α for hexadecane on **ePDMS**_**DMS**_ was lowered compared to fluorinated brush counterparts, as both the **PDMS** substrate and polymerized brushes of **DMS** were readily swelled by hexadecane, leading to enhanced lubrication and dynamic dewetting behavior.^24,26^ In contrast, the swelling of grafted fluorinated brushes was inhibited and resulted in a higher sliding angle for **ePDMS**_**FAS**_ and **ePDMS**_**Mix**_. As swelling of the brushes was impeded, this designates an opposing trend in solvent effects usually observed in LLS, where organic solvents swell the grafted brushes to create a “blended liquid–liquid interface”.^24^ On hard and polar substrates where polymer brushes are present as a thin layer followed by a dissimilar chemical composition, this leads to greatly improved droplet mobility. However, this constitutes an undesirable process for **PDMS** substrates, as the probing liquid would persistently cause swelling of the elastomer and ingress into the substrate. Therefore, blending low-surface-energy groups into the polymer brushes allowed for good droplet mobility without compromising the underlying silicone substrate.

**Table 2:** Sliding Angles (α) for Diiodomethane, Hexadecane (Hex), and PEG-200

Sample	α_CH_2_I_2__	α_Hex_	α_PEG-200_
ePDMS_DMS_	9.8 ± 1.2°	12.8 ± 2.9°	>30°
ePDMS_FAS_	11.8 ± 0.7°	21.8 ± 1.1°	23 ± 1.2°
ePDMS_Mix_	5.8 ± 1.1°	17.8 ± 1.7°	19 ± 0.7°
ePDMS	14.8 ± 1.0°	15.3 ± 0.8°	>30°

Contact angle goniometry showed increased static contact angles (θ) of the testing liquids after functionalization of **ePDMS** with fluorinated silanes due to unfavorable interactions with low surface tension fluorinated groups.^50^ For comparison, silane coatings were also applied to glass substrates. Encouragingly, the observed trend of increasing θ, lowered Δθ, and calculated γ_sv_ was similar for both silanized **ePDMS** and silanized glass substrates, indicating the applicability of this approach to both hard and soft substrates (Tables S3–S5).

**Figure 5:**
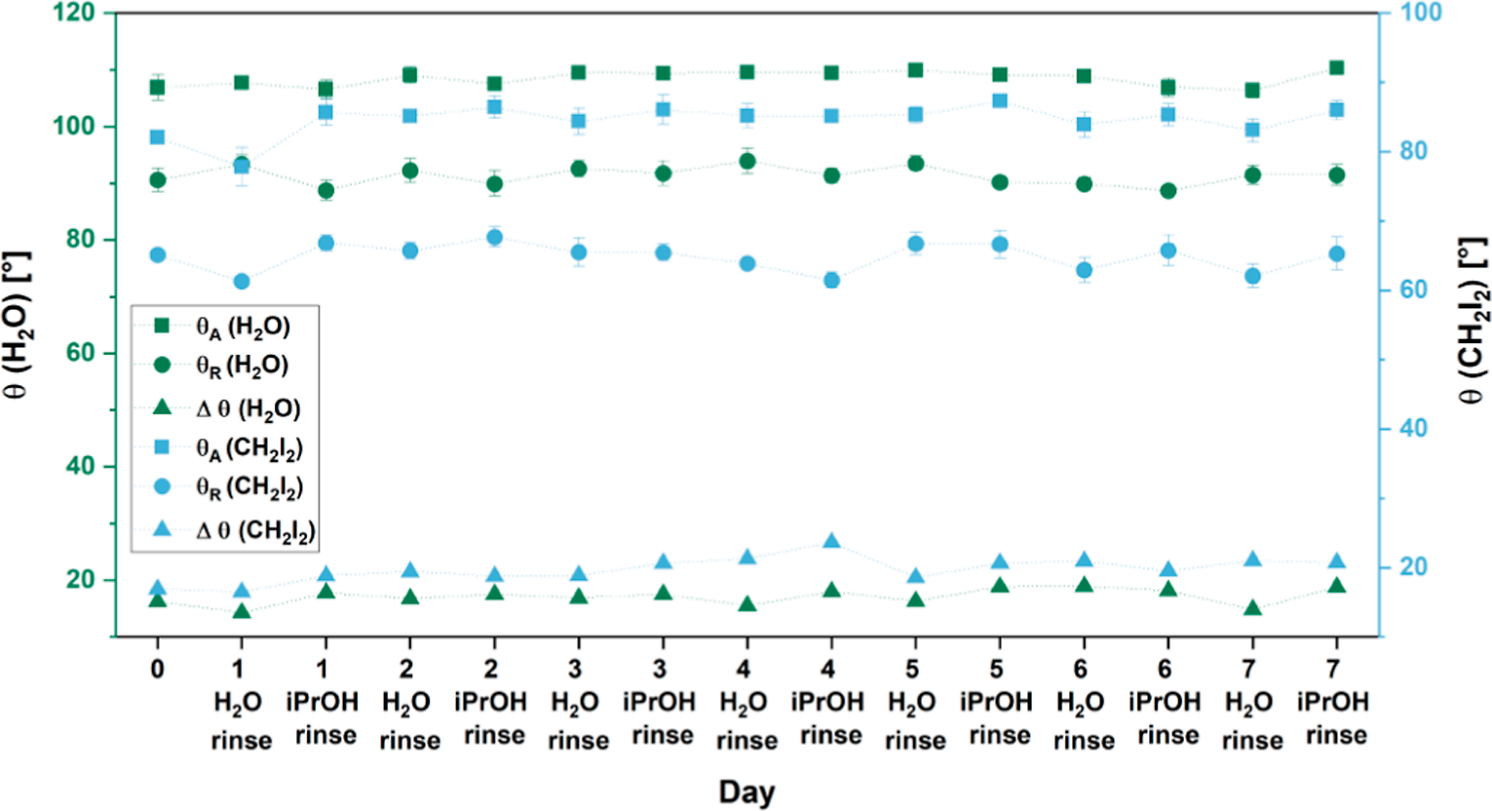
Dynamic contact angles of water and diiodomethane on **ePDMS**_**Mix**_ over 7 daily cycles of rinsing with water and isopropanol.

To address the stability of the functionalization over time, we undertook a daily rinsing cycle of **ePDMS**_**Mix**_ with water and isopropanol (representing an aqueous and an organic solvent) and monitored the dynamic contact angles for water and diiodomethane over 7 days (Figure 5). The liquid-like properties of the functionalization remained stable, as no significant change in Δθ was found.

### 2.3 Effect of Activation and Functionalization on Optical Properties of Functionalized Substrates

**Figure 6:**
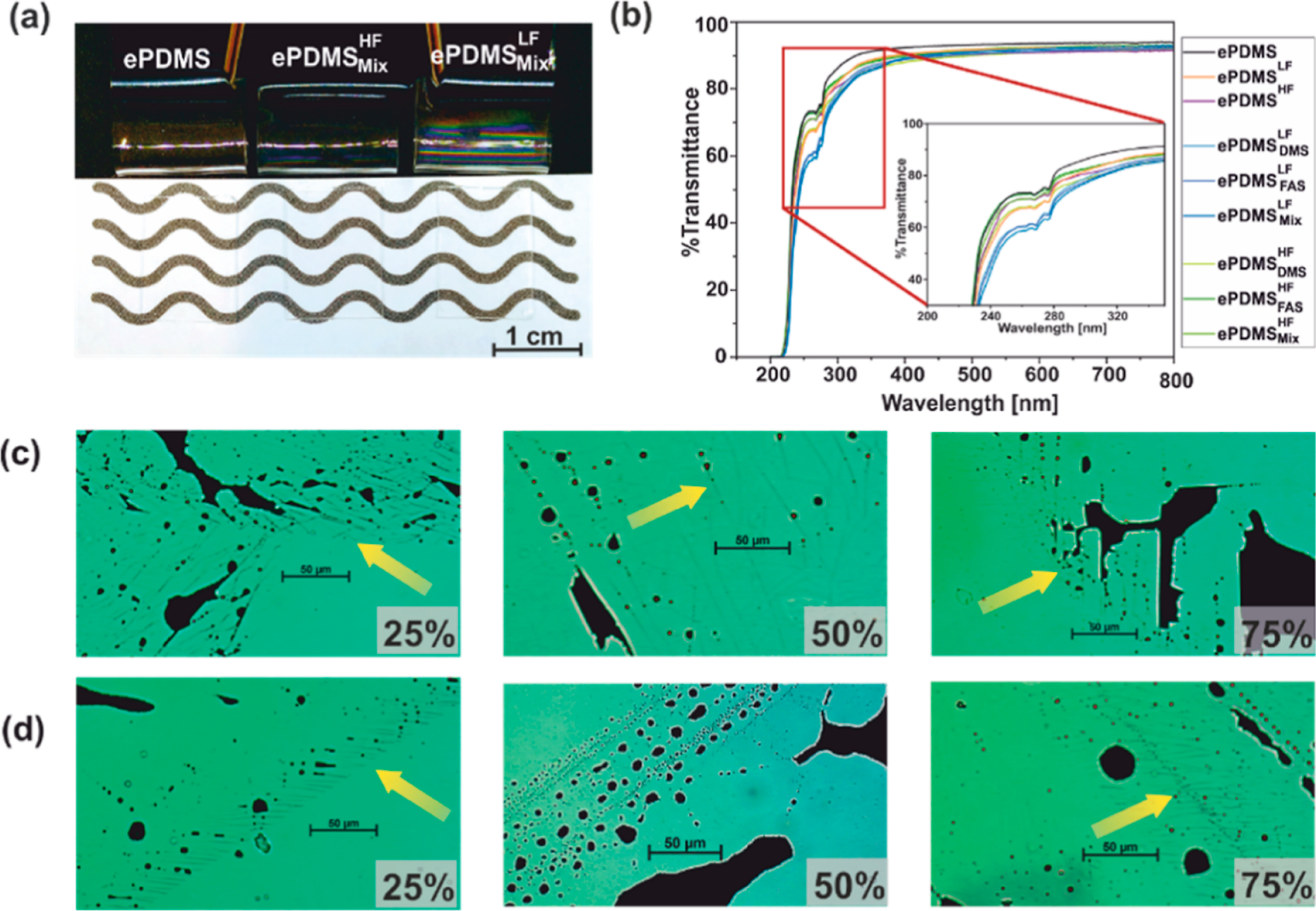
(a) Bending of samples reveals thin-film iridescence on **ePDMS**^**LF**^_**Mix**_. Samples remain transparent after activation and coating. (b) Light transmittance of **ePDMS** substrates. (c) Optical microscopy of **ePDMS**^**LF**^_**Mix**_ reveals superficial cracking at all power settings, highlighted by ink infiltration. (d) Optical microscopy of **ePDMS**^**HF**^_**Mix**_ shows decreased and finer cracking for 25 and 75% power compared to that in (c). No cracking was found on samples activated at 50% power.

Coatings and materials transparency are important for any applications requiring light transmission, such as for optoelectronic displays,^51,52^ solar harvesters, or transparent release films in manufacturing. **PDMS** is transparent in the UV–vis region above 280 nm,^3,53^ with partial transmittance in the region between 240 and 280 nm. Plasma activation led to a slight decrease in transmittance in **ePDMS**. For all functionalizations (**DMS**, **FAS**, and **Mix**) on **ePDMS**, transmittance reduction by approximately 6% for LF-plasma-activated **ePDMS** (**ePDMS**^**LF**^) and 3% for HF-plasma-activated **ePDMS** (**ePDMS**^**HF**^) occurred mainly in the range from 280 to 400 nm (Figure 6b). However, the impact of LF versus HF plasma can be more clearly observed in the 240–280 nm region, as all **ePDMS**^**LF**^ samples showed a transmittance lower than that of **ePDMS**^**HF**^ samples. Above 400 nm, transparency differences for both activation frequencies are negligible, and samples appeared to visually retain their transparency after surface activation and functionalization (Figure 6a).

The **ePDMS**^**LF**^ additionally exhibited superficial iridescence (Figure 6a) when viewed during mechanical deformation. This iridescence was indicative of a glassy, silica-like layer introduced by plasma activation, with a thickness in the range of the wavelength of visible light, which is in good accordance with previously reported thicknesses of the silica-like surface layer after plasma treatment.^39,54^ Interestingly, for **ePDMS**^**HF**^, the iridescence was distinctly decreased.

The mismatch in elastic moduli between the glassy layer and the elastomer bulk can result in spontaneous cracking of the former.^37,54^ This phenomenon was indeed observed in some of our plasma-activated samples. It should be noted that, unlike for previous studies,^37,38,41^ the activated specimens were purposely subjected to severe mechanical deformation, e.g., **PDMS** folding on itself by 180°, to deliberately cause surface cracking of the silica-like layer. Optical microscopy showed prominent cracking for all **ePDMS**^**LF**^ (Figure 6c). In contrast, for **ePDMS**^**HF**^, significantly decreased cracking and reduced crack prevalence was observed at all tested power settings, with no cracking observable for **ePDMS**^**HF**^_**Mix**_ activated at 50% power (Figure 6d). To aid in crack visualization, the sample surfaces were marked with a waterproof ink. Subsequent rinsing with acetone revealed residual ink visible in the surface cracks on samples activated with LF plasma, while no ink retention could be observed on samples activated with HF plasma (Figure S6).

### 2.4 Effect of Extraction and Functionalization on Mechanical Properties

**Figure 7:**
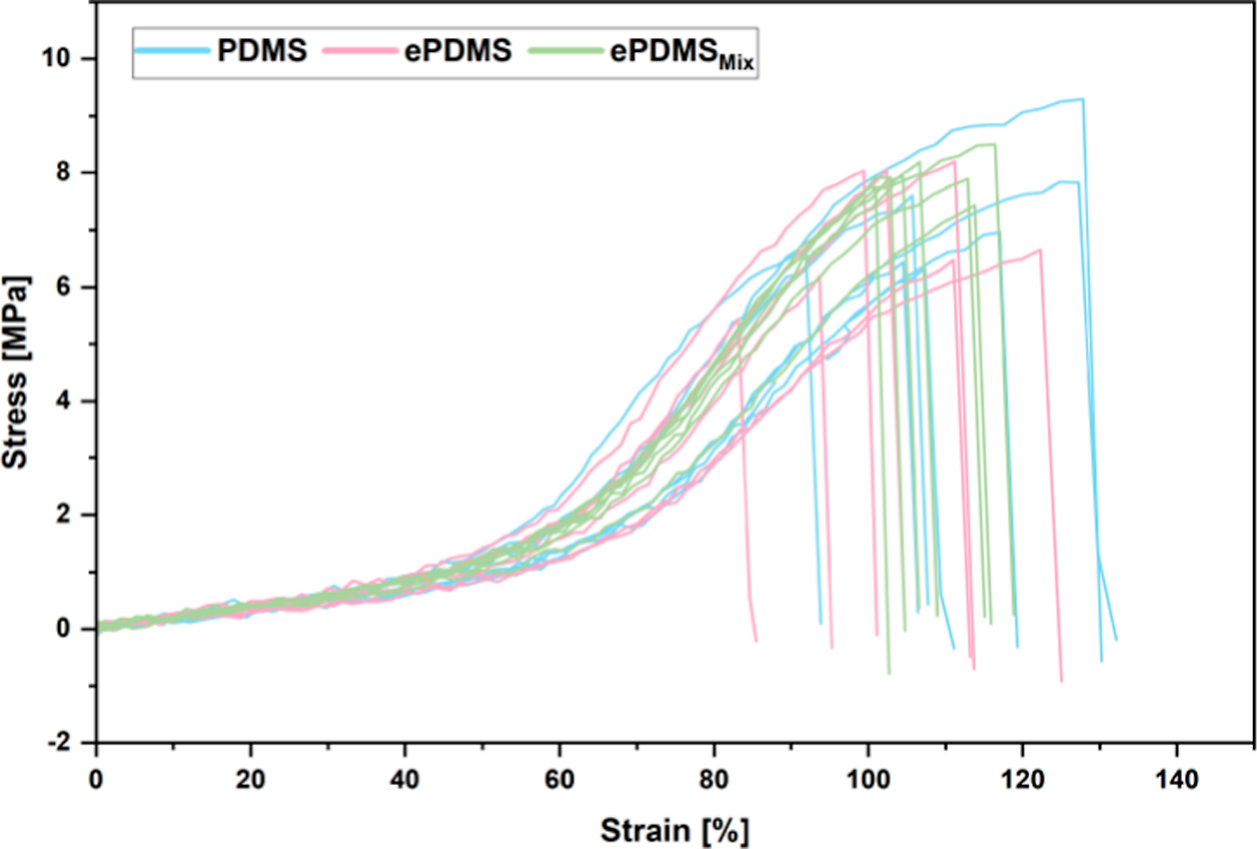
Overlaid stress–strain curves for **PDMS** (blue), **ePDMS** (pink), and **ePDMS**_**Mix**_ (green).

As the extraction, activation, and functionalization processes might impact the mechanical properties of **PDMS**, we conducted tensile tests according to an adapted norm for mechanical testing of elastomers (ASTM D412). Dogbone-shaped test specimens were stamped out from PDMS sheets and strained until failure at 500 mm/min. We found similar stress–strain profiles for all test series (Figure 7) and obtained the ultimate tensile strength for **PDMS**, **ePDMS**, and **ePDMS**_**Mix**_ at 7.2 ± 1.0 MPa, 7.0 ± 1.1 MPa, and 7.9 ± 0.3 MPa, respectively. Given the standard deviation, this indicates no loss of tensile strength throughout the **PDMS** modification and functionalization process. Additionally, the elastic moduli were obtained, yielding 1.60 ± 0.28 MPa for **PDMS**, 1.70 ± 0.28 MPa for **ePDMS**, and 1.85 ± 0.12 MPa for **ePDMS**_**Mix**_ and showing a marginal increase in sample stiffness.

### 2.5 Surface Composition of Functionalized Substrates

**Figure 8:**
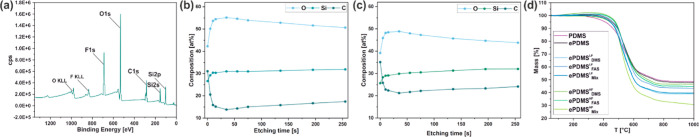
(a) Elemental survey of **ePDMS**^**HF**^_**Mix**_. (b) Etching profile for **ePDMS** activated with LF plasma shows higher oxygen content and less carbon overall. (c) Etching profile for **ePDMS** activated with HF plasma shows a lower disparity between oxygen and carbon at %. (d) Thermal decomposition curves for unaltered, extracted, and extracted and functionalized **PDMS** under a nitrogen atmosphere.

We analyzed the surface elemental composition of substrates via X-ray photoelectron spectroscopy (XPS) and conducted Ar-ion etching with serial etching steps to gain depth information. Since silanes for functionalization and bulk **PDMS** all contain Si, C, and O, we selected F as a marker for successful formation of **ePDMS**_**Mix**_ and **ePDMS**_**FAS**_ substrates. XPS survey spectra indeed confirmed the presence of fluorine in **ePDMS**^**HF**^_**FAS**_, **ePDMS**^**LF**^_**FAS**_, **ePDMS**^**HF**^_**Mix**_, and **ePDMS**^**LF**^_**Mix**_ (Figures 8a and S7).

When only the effect of plasma activation was investigated, elevated oxygen content was observed for **ePDMS**^**LF**^ over the entire etching experiment (255 s total etch time), as evident from the increase in atomic percent (at %) of oxygen at the expense of carbon (Figure 8b), which is indicative of the formation of a silica-like layer.^42,55^ In contrast, **ePDMS**^**HF**^ exhibited lower oxygen incorporation and a residual carbon content of about 20 at % or higher (Figure 8c). The silicon content was not affected by the activation plasma frequency. Additionally, deconvolution of elemental scans for silicon showed organic silicon in the range of 101.7 eV and SiO_2_-species at approximately 103.0 eV.^56^**ePDMS**^**HF**^ exhibited an elevated organic silicon content in comparison with **ePDMS**^**LF**^ (Figure S11).

Carbon elemental scans feature multiple carbon species with binding energies ranging from 284.8–294 eV, which were assigned to C–C bonds at 284.8 eV, adventitious carbon species C–O–C at 286 eV, O–C=O at 288.5 eV, CF_2_-groups at 292 eV, and CF_3_-groups at 294 eV.^57^ Since **FAS** carries two CH_2_- and five CF_2_-groups and one terminal CF_3_-functionality in the side chain, an area ratio of 1:5 for the signals at 294 eV for CF_3_-groups and 292 eV for CF_2_-groups would be expected for functionalization containing **FAS**. Area ratios of 1:4.9 for **ePDMS**^**LF**^_**Mix**_, 1:6.3 for **ePDMS**^**LF**^_**FAS**_, 1:10.4 **ePDMS**^**HF**^_**Mix**_, and 1:10.9 **ePDMS**^**HF**^_**FAS**_ were calculated from XPS peak deconvolution (Figures S9 and S10). As the C 1s signal at 284.8 eV was more prevalent in HF than in LF-plasma activated **ePDMS**, the peak ratio for CF_2_- and CF_3_-groups was distorted by this stronger carbon peak, which explained the ratio deviation from expectation.

### 2.6 Thermal Decomposition of Liquid-like Brushes

Thermogravimetric analysis was carried out to confirm the extraction process and successful functionalization of **ePDMS** (Figure 8d). The thermogram of **PDMS** shows an initial mass loss at 225 °C due to the decomposition of LMW species. In the case of **ePDMS**, the initial mass loss occurred later at 400 °C, which confirmed the successful removal of LMW components. As LLS were prepared from **ePDMS**, thermal analysis showed that no volatile species were lost at lower temperatures but a lower residual mass was found for all functionalized samples, regardless of the activation conditions. This is consistent with the presence of non-cross-linked, linear chains tethered to the surface, since minimally or non-cross-linked **PDMS** fully decomposes during thermal analysis as volatile cyclic oligomers are formed and no residual mass is observed.^58^ The lack of cross-linking in superficial linear polymer brushes allows molecular motion and siloxane chain cyclization, as well as the subsequent elimination of volatile products. Furthermore, the polymer brushes grafted to **ePDMS** are on the external surface, thereby having an increased tendency of volatilization instead of entrapment and conversion to residue within the bulk **PDMS**. The lower residual mass for the functionalized samples supports the observation of superficial functionalized layers, in line with XPS/ion-etching experiments.

### 2.7 Solid Adhesion in Horizontal Push Tests

**Figure 9:**
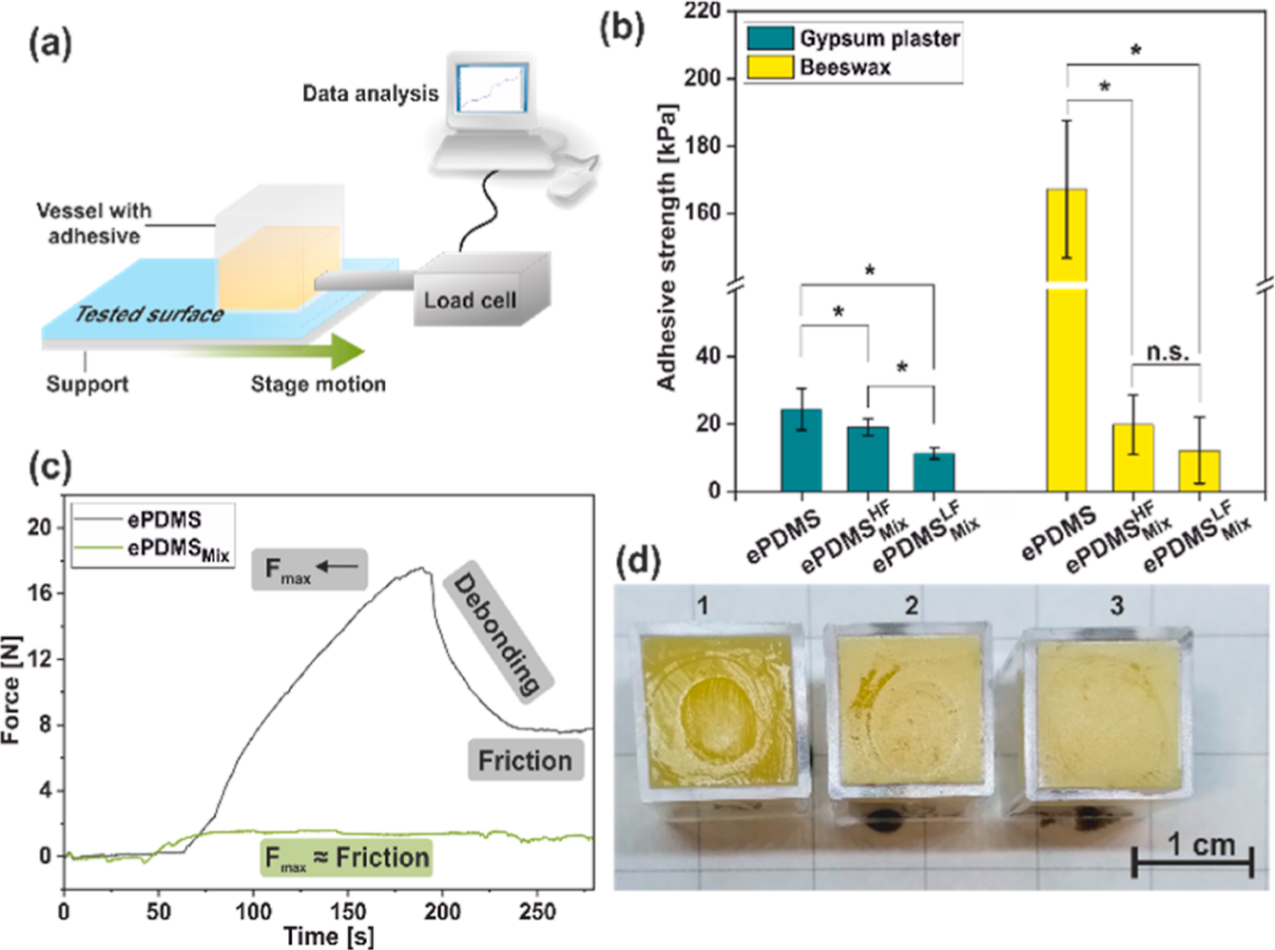
(a) Schematic illustration of the adhesion test configuration. (b) Solid adhesion on **ePDMS**_**Mix**_ and bare **ePDMS**. Data was subjected to one-way ANOVA, *p* = 0.05, * = statistically significant difference, n.s. = not significant. (c) Force profiles for beeswax on **ePDMS** and **ePDMS**^**HF**^_**Mix**_. (d) Beeswax interfaces after separation in adhesion testing on **ePDMS** (1), **ePDMS**^**LF**^_**Mix**_ (2), and **ePDMS**^**HF**^_**Mix**_ (3).

Besides contact angle measurements, which probe liquid–solid interactions, we evaluated the adhesion between the functionalized **PDMS** substrates and solid contaminations. Solid adhesion was determined via a modified tensile testing system to allow for a shear-based (Mode II) horizontal push test configuration (Figure 9a). We selected materials that started off as fluids and subsequently solidified to maximize molecular contact at the testing interface. Gypsum was chosen as a representative sample of an inorganic material, and beeswax was chosen as a representation of an organic, biologically derived material. Detailed information on the test configuration can be found in the Supporting Information. Generally, the force curves on **ePDMS** describe an elastic region followed by plastic deformation and finally adhesive failure, resulting in interfacial debonding for gypsum plaster and solidified beeswax. This relationship is usually observed when examining lateral friction and adhesion of solids^59^ as well as liquids^60^ on solid surfaces. On **ePDMS**_**Mix**_, adhesion force curves of gypsum plaster showed comparable characteristics with plain **ePDMS**, albeit with reduced forces (Figure S14). However, the force profiles for beeswax on **ePDMS**_**Mix**_ did not show distinct debonding peaks but rather suggest that the initial force required to debond beeswax roughly corresponds to the kinetic friction force across the surface (Figure 9c).^60,61^ The **ePDMS**_**Mix**_ substrate exhibited reduced adhesive strength irrespective of plasma activation frequency in the case of beeswax, whereas for gypsum plaster, the adhesive strength was lowered for **ePDMS**^**LF**^_**Mix**_ versus **ePDMS**^**HF**^_**Mix**_, which we attribute to a superficial smoothing effect due to the more prominent glassy layer (Figure 9b). Interestingly, in the case of beeswax, improved flow of melted beeswax across the **ePDMS**_**Mix**_ surfaces could be qualitatively observed. Beeswax that contacted unfunctionalized **ePDMS** showed clear circles from pouring it in liquid form during sample preparation (Figure 9d). We hypothesize that these concentric rings originate from the impeded flow of liquid wax when it contacts unfunctionalized **ePDMS**, and upon further pouring, new, larger rings formed. The patterning of the beeswax is still visible for **ePDMS**^**LF**^_**Mix**_, albeit to a lesser extent, while only minimal ring patterning is noticeable for **ePDMS**^**HF**^_**Mix**_. This is indicative of a qualitatively enhanced flow behavior of viscous liquids on the prepared surfaces. Additionally, this could point toward an altered heat conduction mechanism on functionalized samples due to superficial polymer brushes.

### 2.8 Evaluation of Ice Adhesion

**Figure 10:**
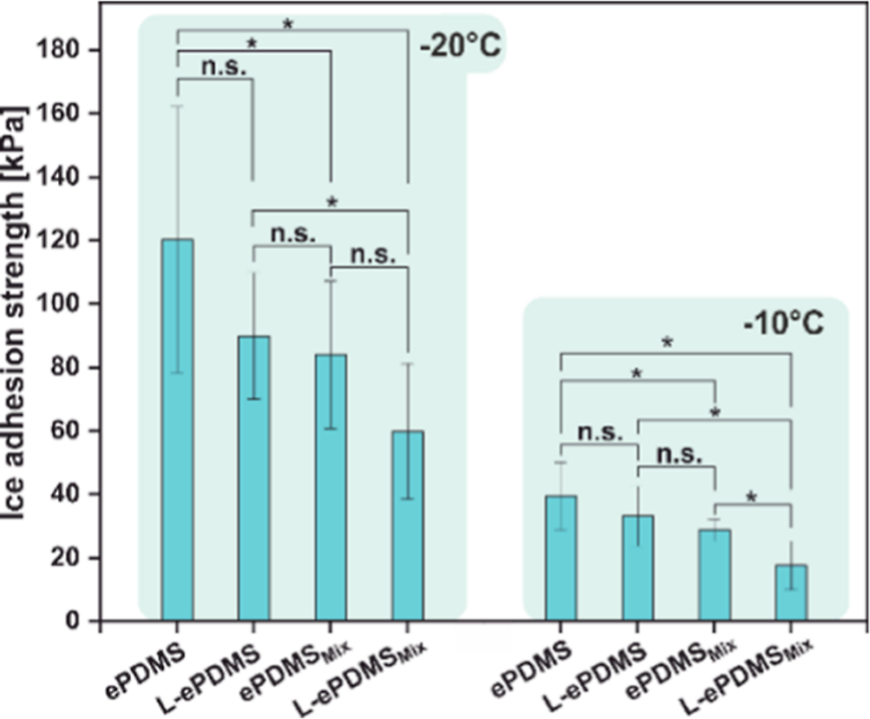
Ice adhesion on bare **ePDMS** and **ePDMS**_**Mix**_ and lubricated substrates at −10 and −20 °C. Data was subjected to one-way ANOVA, *p* = 0.05, * = statistically significant difference, n.s. = not significant.

One of the most important applications of anti-adhesion coatings is the generation of ice-phobic surfaces, as ice accretion poses a ubiquitous problem in particular for aerial and marine vehicles, as well as for power, communications, and general infrastructure in terms of safety hazards and lowered performance efficiency. **PDMS** has been extensively studied for ice-phobic coatings, not just because of its well-known hydrophobicity and low surface energies but also due to the modulus mismatch between stiff ice and elastomeric **PDMS**, which has been shown to induce cavitation at the interface and aid ice-delamination.^62^ We therefore extended our studies to test the anti-icing properties of preoptimized **ePDMS**^**HF**^_**Mix**_ using horizontal push tests. Additionally, polymer brush surfaces were lubricated to investigate the impact on icing. Tests were conducted at −20 and −10 °C. At both temperatures, ice adhesion was reduced by a third for **ePDMS**_**Mix**_ compared to **ePDMS**. Lubrication of **ePDMS**_**Mix**_ by soaking the functionalized elastomer in a perfluoropolyether lubricant (Krytox GLP105) and then wiping off the excess lubricant (referred to as **L-ePDMS**_**Mix**_) led to halving of the ice adhesion strength compared to that of plain **ePDMS** (Figure 10 and Table 3). It should be noted that simply soaking **ePDMS** in Krytox GLP105 did not lead to lubrication, as lubricant dewetting occurred for the **ePDMS** surface. In contrast, the lubricant fully wetted **ePDMS**_**Mix**_ (Figure S1), suggesting that surface/subsurface functionalization is necessary to enable infiltration of the low surface tension lubricant. Further, as stick–slip-like motion of ice was observed on **ePDMS** as well as lubricated **ePDMS** (Figure S15a,c), we conclude that surfaces were not sufficiently lubricated. In contrast, smooth sliding was facilitated on **ePDMS**_**Mix**_ and **L-ePDMS**_**Mix**_ (Figure S15e,g). Both **ePDMS**_**Mix**_ and **L-ePDMS**_**Mix**_ showed ice-phobicity at −20 and −10 °C, as they exhibited ice adhesion strength <100 kPa.^59^ Ice adhesion on **L-ePDMS**_**Mix**_ at −10 °C decreased by approximately three times compared to that at −20 °C, with **L-ePDMS**_**Mix**_ showing ultralow ice adhesion (<20 kPa), which allows for passive removal of ice on moving parts or by gusts of wind,^63^ indicating the potential utility of these functionalized coatings in combination with electrothermal deicing systems. The decreased adhesion at higher temperatures is in line with the known temperature-dependent adhesion behavior of **PDMS** surfaces.^64^ For our functionalized/functionalized and lubricated **ePDMS** specimens, we assume that the decreased ice-adhesion occurred due to the decreased stiffness of the **PDMS** substrate as well as increased mobility of the liquid-like surface and the lubricant at −10 °C versus −20 °C. This was additionally affirmed by the respective force profiles, as sliding and stick–slip motion was observed at −10 °C, while a distinct peak for interfacial debonding and minimal friction was observed at −20 °C (Figure S15a–d).

**Table 3:** Ice Adhesion Strength on the Investigated Substrates at −10 and −20 °C

	Temperature	Ice adhesion strength [kPa]
–20 °C	**ePDMS**	120.2 ± 41.9
	L-ePDMS	89.9 ± 20.0
	**ePDMS**_**Mix**_	84.0 ± 23.2
	L-ePDMS_Mix_	59.9 ± 21.3
–10 °C	**ePDMS**	39.4 ± 10.8
	L-ePDMS	33.2 ± 9.5
	**ePDMS**_**Mix**_	28.8 ± 3.5
	L-ePDMS_Mix_	17.5 ± 7.7

## 3 Conclusions

We were able to overcome the major challenges in **PDMS** activation of fast hydrophobic recovery and glassy-layer formation by substrate extraction and optimized plasma activation. This resulted in chemically homogeneous, slippery surfaces and preserved the optical properties of the specimen. High transparency and self-cleaning behavior pose benefits, for instance, in energy applications, such as solar cell coatings.^51^ Superficial cracking was minimized as silica-like layer formation was suppressed, and mechanical surface homogeneity and uniform stability were preserved, which could allow the deposition of homogeneous films for flexible sensors. Our report focused on preparing liquid-like surfaces comprising siloxane-based polymer brushes on elastomeric **PDMS** by applying the grafting-from concept. As we introduced superficial polymers postcuring, the set mechanical properties of the established formulations were not compromised. Different decomposition mechanisms allowed the confirmation of surface polymer brushes even when the chemical composition was comparable. Their slippery character was enhanced by the addition of a molecular linker in our **Mix** coating. Proof-of-concept testing showed excellent repellency of solid contaminants and revealed the potential for anti-icing applications as ultralow ice adhesion strengths were obtained, which allow for the passive removal of accumulating ice. Such qualities also prove valuable in reducing scaling,^17^ improving flow in **PDMS**-based devices,^65^ and minimizing biofouling,^66^ the latter of which especially poses a significant challenge on **PDMS** surfaces.^11,67^ We anticipate that minimized superficial cracking and simple surface modification to obtain slippery interfaces can alleviate the current constraints in **PDMS** applications.

## 4 Experimental Section

### 4.1 Materials

Detailed information and sections regarding materials, characterization methods, evaluation of solid adhesion, and ice adhesion strength are provided in the Supporting Information.

### 4.2 Preparation of PDMS Elastomer Substrates

To prepare silicone substrates, Sylgard 184 silicone elastomer base was mixed with the respective curing agent in a 10:1 ratio by weight, followed by mechanical stirring for 1 min until both components were homogeneously combined. The mixture was degassed under reduced pressure, poured into a glass Petri dish, and degassed again. The prepolymer was cured in an oven set to 100 °C for 70 min.

#### 4.2.1 PDMS Extraction

To evaluate maximum mass loss, **PDMS** elastomer sheets were demolded from the Petri dish, cut into distinguishable sections, and extracted by immersion of **PDMS** samples (ranging from 0.1 to 0.5 g) in either 20 mL of toluene for 24 h or in 20 mL each of a series of solvents (hexane 24 h, ethyl acetate 24 h, and acetone 48 h). For mass loss profiles, **PDMS** elastomer sheets were cut into distinguishable sections and immersed in either 20 mL of toluene or ethylene glycol for 24 h. To obtain a time profile, the change in mass was monitored at 1, 2, 4, and 24 h intervals of immersion as well as after drying. In all extraction experiments, the solvent was changed once after 2 h when the pieces had swelled completely. **ePDMS** was dried in an oven set to 90 °C until a constant weight was reached.

For utilization for coating, **PDMS** sheets were extracted prior to being cut to size. Full sheets were stirred for 24 h in 100 mL of toluene per g of cured elastomer and dried to a constant weight at 90 °C for 16 h. Rectangular pieces (width = 16 mm, varying length of 40–80 mm) were cut from the sheets as samples and utilized for coating.

#### 4.2.2 Substrate Activation and Functionalization

Substrates were activated with air plasma generated in a Plasma Diener Atto System (0–200 W/40 kHz (LF) or 0–300 W/13.56 MHz (HF)) at reduced pressure (0.14 mbar) and different power settings (25, 50, and 70%) for 60 s. Activated substrates were coated by either applying the coating solution with a pipet to fully cover the surface, followed by tilting the substrate, or dipping the substrate in the coating solution and withdrawing after 10 s. The coating solutions were adapted from previous research.^16^ Briefly, a solution of 1 part silane in 10 parts isopropanol by weight was prepared in a plastic container, and 0.1 part by weight of sulfuric acid respective to isopropanol was added for hydrolysis. For **Mix** coating solutions, the silane portion of the mixture (comprising 1/11 parts of the total mixture or 1:10 respective to the solvent isopropanol) comprised equal weights of dimethoxydimethylsilane and 1*H*,1*H*,2*H*,2*H*-perfluorooctylmethyldimethoxysilane. The solution was allowed to stand for at least 30 min prior to use. After coating, samples were dried for 20 min at room temperature and ambient humidity, followed by rinsing with deionized water, isopropyl alcohol, and toluene and drying to weight consistency.

#### 4.2.3 Fabrication of Lubricated Substrates

To create a lubricated interface on **ePDMS** and **ePDMS**_**Mix**_, 100 μL of Krytox GLP105 was pipetted onto the surface of the substrates. Lubricant infiltration was performed for at least 4 h. Excess lubricant was allowed to drain by placing samples vertically. Lubricant residue was removed by wiping with paper towels until the surface was free of excess lubricant.

## Supporting Information Available

Additional experimental procedures; materials and methods; additional experimental data of extraction test, contact angle, and XPS analysis; adhesion testing; and photographs of the testing setup (PDF)
