# Supplementary material for: Transparent PDMS Surfaces with Covalently Attached Lubricants for Enhanced Anti-adhesion Performance
Source: ACS Appl Mater Interfaces. 2024 Feb 13;16(8):10942–52. doi: 10.1021/acsami.3c17110 (PMC10910447; doi:10.1021/acsami.3c17110)
Supplement: Supplementary file 1 — am3c17110_si_001.pdf [file am3c17110_si_001.pdf]

Supporting Information

Transparent PDMS Surfaces with Covalently Attached Lubricants for Enhanced Anti-Adhesion Performance

Tanja Eder,[a,b] Andreas Mautner,[b,c] Yufeng Xu,[a] Michael Reithofer,*[d] Alexander Bismarck,*[b,e] Jia Min Chin*[a]

[a] Department of Functional Materials and Catalysis, University of Vienna, Währinger Straße 42, 1090 Vienna, Austria
[b] Institute of Materials Chemistry and Research, University of Vienna, Währinger Straße 42, 1090 Vienna, Austria
[c] Institute of Environmental Biotechnology, University of Natural Resources and Life Sciences (BOKU), Konrad-Lorenz-Straße 20, 3430 Tulln a.d. Donau, Austria
[d] Institute of Inorganic Chemistry, University of Vienna, Währinger Straße 42, 1090 Vienna, Austria
[e] Department of Chemical Engineering, Imperial College London, South Kensington Campus, London, SW7 2AZ, UK.

*Corresponding authors:
Michael Reithofer - Institute of Inorganic Chemistry, University of Vienna, Währinger Straße 42, 1090 Vienna, Austria; E-mail: michael.reithofer@univie.ac.at
Alexander Bismarck - Institute of Materials Chemistry and Research, University of Vienna, Währinger Straße 42, 1090 Vienna, Austria; Department of Chemical Engineering, Imperial College London, South Kensington Campus, London, SW7 2AZ, UK; E-mail: alexander.bismarck@univie.ac.at
Jia Min Chin - Department of Functional Materials and Catalysis, University of Vienna, Währinger Straße 42, 1090 Vienna, Austria; E-mail: jiamin.chin@univie.ac.at

S1. Supporting Materials and Methods

S1.1. Materials

Sylgard 184 silicone elastomer (Dow Corning) and Krytox GLP105 (Chemours) were purchased from ULBRICH Maschinenbau- und Export- Import Betriebs G.m.b.H. 1*H*,1*H*,2*H*,2*H*-Perfluorooctylmethyldimethoxysilane (96%) was obtained from abcr. Dimethoxydimethylsilane (95%) and diiodomethane (99%) were obtained from Sigma-Aldrich. Acetone (99.5%) was obtained from Thermo Fisher. Isopropanol and Polyethylene glycol (PEG-200, MW ~ 200 g/mol) were obtained from VWR Chemicals. Toluene (<99.9%) was obtained from Carl Roth. Hexadecane (<98%) was obtained from TCI Chemicals. Ethyl Acetate (99.5%) and n-Hexane (<97%) was obtained from Honeywell. Gypsum plaster was obtained from a local hardware store (OBI Bau- und Heimwerkermärkte GmbH). Glass microscope slides were purchased from Labsolute. Polystyrene cuvettes for adhesion testing were obtained from LLG. All materials were used without any further purification.

S1.2. Methods

S1.2.1. Adhesion Testing via Horizontal Push Tests

Samples for adhesion testing were prepared as follows: 5 g Sylgard 184 mixed in a 10:1 base to crosslinker ratio were poured in a petri dish and spread using a spin-coater (SPS SPIN150i-NPP) at low speeds. The prepolymer mixture was spun at 70 rpm with 35 rpm/s spin acceleration for 10 s followed by a wiggle mode with a period of 100 ms, 180° amplitude and maximum speed of 70 rpm for 20 s. The petri dish was subsequently covered and placed on a tilting stage in an oven to ensure a level surface. The mixture was allowed to self-level for an additional 30 min at room temperature and then cured at 100°C for 70 min. Cured **PDMS** sheets were extracted as previously described, cut into strips of equal width and functionalized (Section **4.2.1.** and **4.2.2.**).

Samples for testing were cut into 16 x 19 mm rectangles and attached to a 20 x 25 mm glass support with double-sided tape. Square polystyrene tubing with 10.4 x 10.4 mm internal cross-sections were fabricated from cut-offs of single-use polystyrene cuvettes and employed as containers for the tested adhesives. Gypsum plaster samples were prepared by stirring one part powder with 0.75 parts deionized water to combine and 0.5 ml filled into the container followed by drying for at least 10 h. Beeswax samples were prepared by melting beeswax at 60-80 °C and poured into aligned cuvettes to afford equal height on all samples, followed by testing within 2 h of sample preparation. To determine ice adhesion, adhesive containers were filled with 600 µl milli-Q water and samples placed in a humidity chamber (Binder MKF 56) at either -10 °C or -20 °C and RH(set) = 50% for at least 16 h.

To determine the adhesive strength, a horizontal push test was performed in a Deben Microtester using a probe attached to a 2 kN load cell fixed on one side and an extendable stage for sample mounting on the other side. The substrate with bonded contaminant was moved towards the probe (**Fig. 6a**) and the force for debonding recorded. An extension speed of 0.5 mm/min and sampling time of 200 ms were selected and a minimum of 6 samples were tested for each adhesive and surface. For ice adhesion horizontal push tests were carried out at either -10 °C or -20 °C with the Deben Microtest set up inside a humidity chamber following the same parameters as solid adhesion testing at room temperature. Adhesive strength was calculated from the maximum force divided by the occupied area of the adhesive. Further, data was subjected to one-way ANOVA and Tukey means comparison test.

S1.2.2. Tensile testing

Tensile testing was conducted in an Instron universal testing machine. ASTM D412 was adapted regarding the specimen sample geometry. **PDMS**, **ePDMS** or **ePDMS**$_{\mathbf{Mix}}$ sheets were stamped to yield dogbone-shaped specimen (75 mm total length with a reduced section of 58 mm x 5 mm). The thickness of the samples was measured individually with calipers and was within 0.55 to 0.70 mm. The samples were fixed in the clamps of the universal testing machine with two layers of cardboard to reduce stress applied by serrated sample clamps and strained until failure at 500 mm/min. Ultimate tensile strength was calculated as the maximum recorded force divided by the area for each test and averaged. The elastic modulus was obtained from the slopes of the linear portion of the stress strain curves for each sample and averaged.

S1.2.3. Contact Angle Analysis

Sessile contact angle measurements were performed on a Krüss Drop Shape Analyzer DSA30S paired with ADVANCE software at 20 °C and ambient humidity. Cleaned substrates were placed on the measuring stage and a 5 µl droplet of liquid was manually dispensed with a pipette. The measurement was started with a 2 s delay for 5 s at 1 fps. Sessile contact angles reported are a mean of at least 5 individual measurements and the standard deviation was below 1.5° unless stated otherwise. The drop shape was fitted by tangent fit. Sessile contact angles were determined using milli-Q water, toluene, diiodomethane, hexadecane, and PEG-200.

Dynamic contact angle measurements were performed on a Krüss Drop Shape Analyzer DSA30S equipped with a software-controlled dosing unit paired with ADVANCE software at 20 °C and ambient humidity. Dynamic contact angles reported are a mean of at least 5 individual measurements and the standard deviation was below 2.5° unless stated otherwise. For simultaneous measurement of the advancing and the receding contact angle, a clean substrate was placed on the measuring stage and a 0.5 µl droplet was dosed and subsequently deposited onto the substrate. After the needle was aligned centrally in the droplet, 10 µl were dosed at 0.05 ml/min and after a 30-second waiting period, 5 µl were dosed at 0.006 ml/min while simultaneously measuring the advancing contact angle at 1 fps. After another 30-second waiting period, the total dosed volume of 15 µl was aspirated while again measuring the receding contact angle at 1 fps. The larger volume for the receding angle measurement was necessary to ensure the measurement is conducted in the correct range[1].

Sliding angles were evaluated by placing the substrates on a tilting stage and applying 10-20 µl of testing liquid. The tilt angle was slowly increased until droplet motion could be registered; simultaneously the contact angle was recorded. Reported sliding angles consist of at least 4 individual measurements and the standard deviation was below 2.0°.

S1.2.4. Thermogravimetric Analysis

For thermogravimetric analysis thin sheets were either cut horizontally from the sample surface to obtain representative thermal decomposition curves on the surface coatings or uniformly sized discs punched out from coated thin films. Analysis was performed on a NETZSCH STA 449 F3 Jupiter under a nitrogen atmosphere (purge at 20 ml/min). Thin sample sheets were placed to lie flat on the bottom of Al_2O_3 crucibles and heated from room temperature to 1000 °C at 10 °C/min.

S1.2.5. Optical Investigations

Transparency was estimated via UV/Vis spectroscopy. Data was collected on an Agilent Cary 60 spectrometer at scans from 200 to 800 nm and a medium scan rate of 600 nm/min and a 1 nm data interval. Absorption spectra were converted to transmission curves via the following relation: Abs-2= -log(%T).

Optical investigation of samples was conducted with a Zeiss Axio Imager 2 at multiple magnifications in transmission mode. For visualization and higher contrast of the surface cracks the prepared substrates were marked with a waterproof marker. Retention of dye was investigated after cleaning of the substrates by rinsing with acetone.

S1.2.6. X-Ray Photoelectron Spectroscopy

XPS data was acquired on a Thermo Scientific Nexsa Photoelectron Spectrometer using Al Kα X-rays as the source. XPS was performed on a spot size of 400 µm with an energy step size of 1 eV for the survey and 0.1 eV for detailed analysis. For composition analysis, the coated ePDMS samples were etched for either 3 x 5 s, 3 x 20 s and 3 x 60s or in ascending intervals of 2 s, 5 s, 10 s, 20 s, 30 s, 60 s and 120 s to gain information about the top layer and depth of modification alike. For data analysis binding energy was calibrated to C_{1s} = 284.8 eV.

S2. Additional Data

S2.1. Lubrication

Preferential wetting for coated **ePDMS** samples compared to **ePDMS** was apparent as lubricant drew away from the **ePDMS** surface, while wetting occurred due to the **Mix** coating. Excess lubricant readily vacated the surface for **ePDMS**, while strong tailing and impeded draining was observed for **ePDMS$_{Mix}$**. Lubricant residue was removed by wiping with paper towels. (Wiping with paper towel proved successful in removing excess according to microscopy images; rinsing with water, ethanol or Novec7200, stirring in acetone or sonication in ethanol showed that excess lubricant remained on the surface).

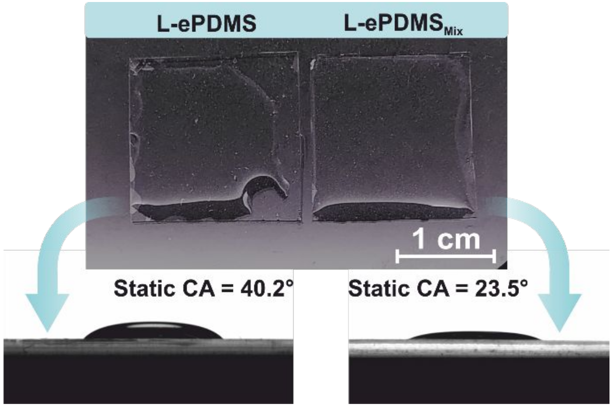

Fig. S1: 100 µl Krytox GLP105 applied to **ePDMS** and **ePDMS$_{Mix}$** and respective static contact angles of the lubricant implying preferential wetting and **SLIPS** formation for **ePDMS$_{Mix}$**

Table S1: Static contact angle for Krytox GLP105 on **ePDMS** and **ePDMS$_{Mix}$**.

	Krytox GLP105
**ePDMS**	40.2 ± 0.80°
**ePDMS$_{Mix}$**	23.5± 1.25°

S2.2. PDMS Extraction

Table S2: Mass loss for Sylgard 184 silicone elastomer upon extraction in toluene and a cascade of solvents with decreasing **PDMS** solubility.

	$m_{Solvents}$ [%]	$m_{Toluene}$ [%]
**Sylgard 184**	-4.6 ± 0.01	-4.6 ± 0.01

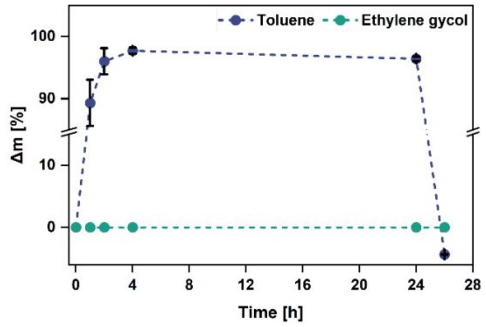

Fig. S2: Mass change profile over 24 h of Sylgard 184 in toluene and ethylene glycol and subsequent drying.

S2.3. Contact Angle Analysis

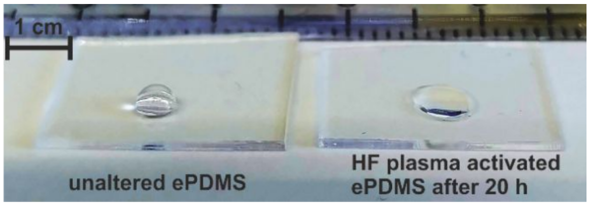

Fig. S3: A 5 µl water droplet placed on **ePDMS** and **ePDMS** 20 h after HF plasma activation.

Table S3: Static contact angles (θ) of common testing liquids on bifunctional silane coatings on **ePDMS**. θ are a mean of at least 5 independent measurements and standard deviation was below 1.5°. No static contact angle for toluene on **ePDMS$_{DMS}$** could be established due to immediate swelling.

Sample	θ_{Water}	$\theta_{Diiodomethane}$	$\theta_{Toluene}$	$\theta_{PEG-200}$	$\theta_{Hexadecane}$
**ePDMS$_{FAS}$**	102.4°	92.2°	64.6°	86.7°	72.1°
**ePDMS$_{Mix}$**	106.9°	85.7°	45.4°	83.1°	58.0°
**ePDMS$_{DMS}$**	104.7°	81.5°	/	87.9°	45.9°
**ePDMS**	100.8°	85.9°	44.6°	92.2°	41.8°

Table S4: Static contact angles (θ) of common testing liquids on bifunctional silane coatings on glass slides. θ are a mean of at least 5 independent measurements and standard deviation was below 1.5°.

Sample	θ_{Water}	$\theta_{Diiodomethane}$	$\theta_{Toluene}$	$\theta_{PEG-200}$	$\theta_{Hexadecane}$
**Glass$_{FAS}$**	100.7°	85.8°	60.2°	79.3°	64.5°
**Glass$_{Mix}$**	98.0°	79.5°	48.1°	75.3°	55.7°
**Glass$_{DMS}$**	99.2°	78.7°	25.9°	80.0°	36.3°

**Glass**	21.5°	54.4°	/	24.2°	28.6°

Table S5: Advancing contact angles (θ_A), receding contact angles (θ_R), contact angle hysteresis ($\Delta\theta$) for water and diiodomethane and OWRK method calculated solid surface tensions (γ_{sv}). Dynamic contact angles reported are a mean of at least 5 individual measurements and the standard deviation was below 2.0° unless stated otherwise. Dynamic water contact angles on glass could not be established via needle-in-drop method.

Sample	θ_{a,H_2O}	θ_{r,H_2O}	$\Delta\theta_{H_2O}$	θ_{a,CH_2I_2}	θ_{r,CH_2I_2}	$\Delta\theta_{CH_2I_2}$	γ_{sv} [mN/m]
**Glass$_{DMS}$**	104.8 ± 0.6°	86.7 ± 0.7°	18.1°	74.4 ± 0.9°	60.5 ± 0.4°	13.9°	20.5
**Glass$_{FAS}$**	103.3 ± 0.6°	80.9 ± 1.3°	22.4°	85.5 ± 1.0°	60.9 ± 0.6°	24.6°	15.5
**Glass$_{Mix}$**	101.2 ± 0.9°	78.3 ± 1.4°	22.9°	73.8 ± 1.6°	62.6 ± 1.3°	11.2°	20.9
Glass	/	/	/	50.7 ± 0.6°	27.3 ± 0.7°	23.5°	/

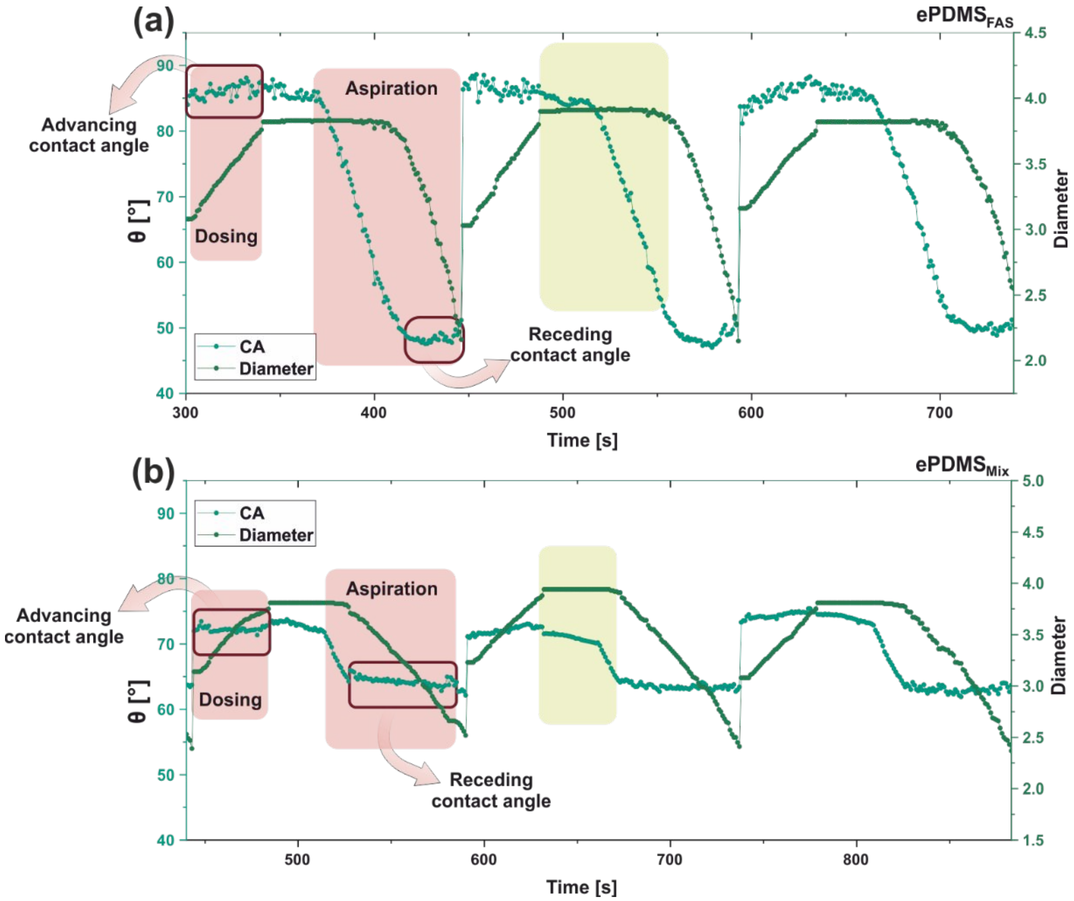

Fig. S4: Reduced droplet baseline pinning of CH_2I_2 in (a) **ePDMS$_{Mix}$** compared to (b) **ePDMS$_{FAS}$**. The plateau in droplet diameter (dark green line) is highlighted in the yellow box. Dosing and aspiration is indicated by the pink box. Red boxes mark the measurement for the advancing and receding contact angle (see also Section **S1.2.2.**).

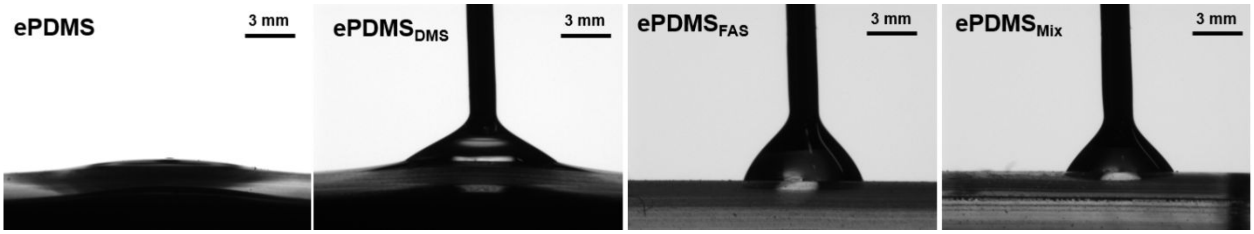

Fig. S5: Swelling of substrates upon contact with hexadecane. Bulging of **ePDMS** and **ePDMS$_{DMS}$** is visible, while **ePDMS$_{FAS}$** and **ePDMS$_{Mix}$** remain completely flat.

Table S6: Initial contact angle data reported in the submitted manuscript. Dynamic contact angle data on **PDMS** and **ePDMS$_{DMS}$** could not be obtained due to immediate swelling of the substrates, designated by "-" in the table.

**Sample**	θ_{a,H_2O}	θ_{r,H_2O}	$\Delta\theta_{H_2O}$	θ_{a,CH_2I_2}	θ_{r,CH_2I_2}	$\Delta\theta_{CH_2I_2}$	$\theta_{a,Hex}$	$\theta_{r,Hex}$	$\Delta\theta_{Hex}$	γ_{sv} **[mN/m]**
**ePDMS$_{DMS}$**	108.0 ± 1.8°	80.0 ± 1.0°	28.0°	66.9 ± 2.0°	50.3 ± 1.1°	16.6°	-	-	-	25.1
**ePDMS$_{FAS}$**	114.6 ± 1.4°	87.0 ± 1.0°	27.9°	85.1 ± 1.6°	47.8 ± 1.5°	37.3°	77.8 ± 1.3°	34.8 ± 1.6°	43.7°	15.0
**ePDMS$_{Mix}$**	112.2 ± 1.0°	93.6 ± 2.0°	18.5°	73.4 ± 1.3°	62.6 ± 1.3°	10.8°	57.1 ± 1.4°	33.6 ± 1.8°	23.5°	21.5
**ePDMS**	113.2 ± 0.6°	75.7 ± 1.6°	37.5°	93.4 ± 0.5°	53.2 ± 0.4°	40.2°	-	-	-	17.3

Table S7: Dynamic contact angles for water and diiodomethane of **ePDMS$_{FAS}$** and **ePDMS$_{Mix}$** samples after over 12 months of storing.

**Sample**	θ_{a,H_2O}	θ_{r,H_2O}	$\Delta\theta_{H_2O}$	θ_{a,CH_2I_2}	θ_{r,CH_2I_2}	$\Delta\theta_{CH_2I_2}$
**ePDMS$_{FAS}$**	118.8 ± 0.9°	77.1 ± 2.0°	41.7°	93.2 ± 1.4°	57.5 ± 1.9°	35.7°
**ePDMS$_{Mix}$**	113.2 ± 0.9°	83.7 ± 1.9°	29.5°	86.3 ± 2.0°	62.6 ± 1.8°	24.0°
**ePDMS**	116.3 ± 0.8°	74.8 ± 2.0°	41.5°	91.0 ± 0.5°	47.3 ± 1.9°	43.7°

S2.4. Optical Properties

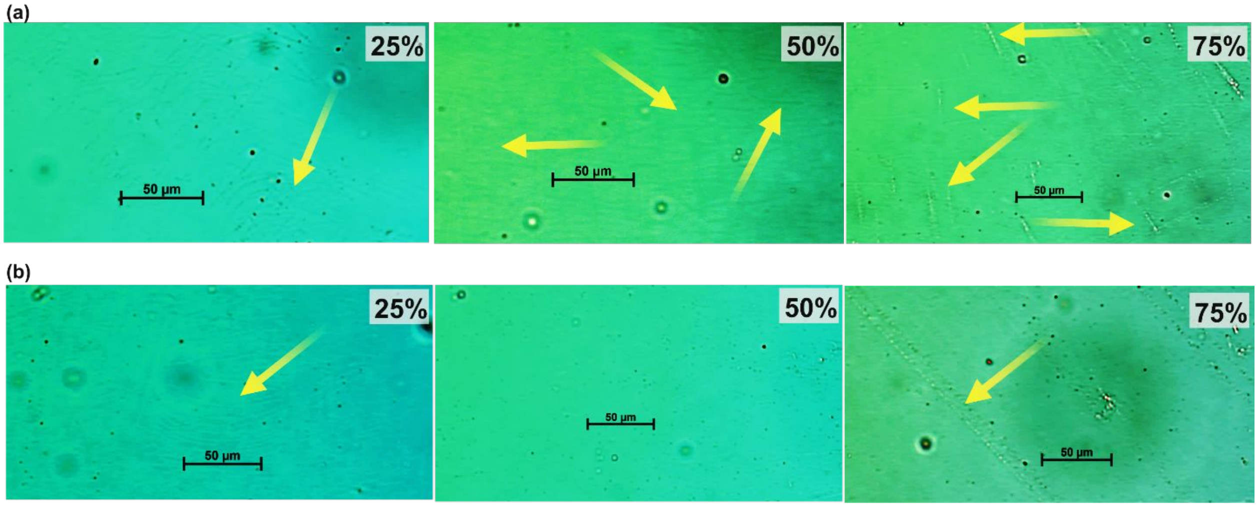

Fig. S6: Optical microscopy images of a) **ePDMS^{LF}$_{Mix}$** and b) **ePDMS^{HF}$_{Mix}$**. Samples were marked with ink for crack visualization, and cleaned with acetone. Cracks for 25% and 50% power of LF plasma appear darker due to ink retention.

S2.5. X-Ray Photoelectron Spectroscopy

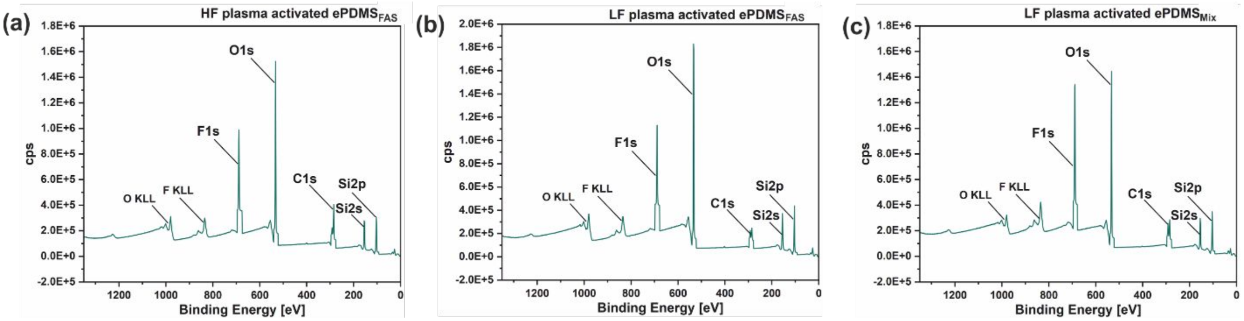

Fig. S7: Elemental survey for (a) **FAS** coating on HF plasma activated **ePDMS** and (b) **FAS** and (c) **Mix** coating on LF plasma activated **ePDMS**. All scans show significant fluorine content on coated substrates.

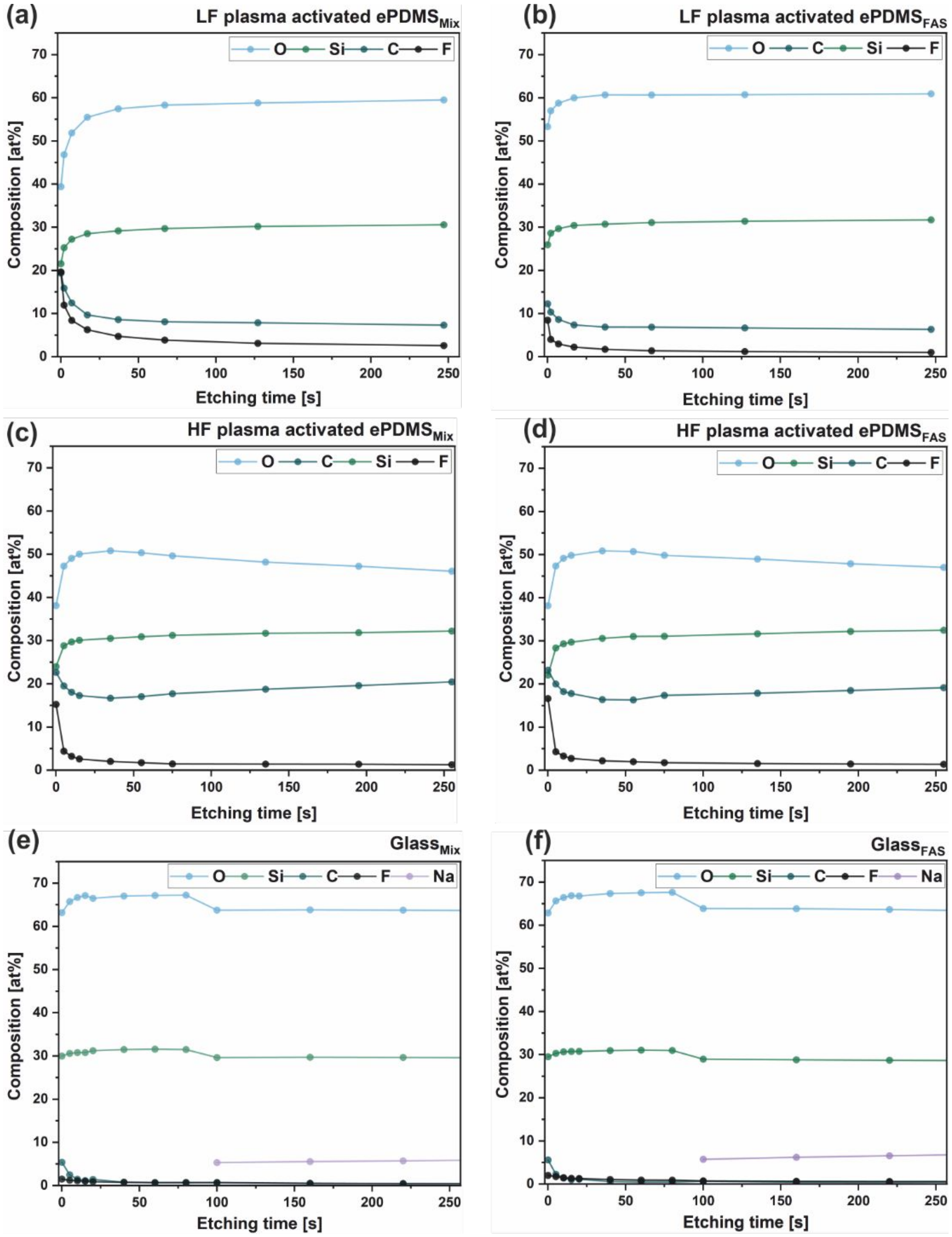

Fig. S8: XPS etching profiles for LF plasma activated (a) **ePDMS$_{Mix}$** and (b) **ePDMS$_{FAS}$**, HF plasma activated (c) **ePDMS$_{Mix}$** and (d) **ePDMS$_{FAS}$** as well as (e) **Mix** functionalized and (f) **FAS** functionalized glass substrates.

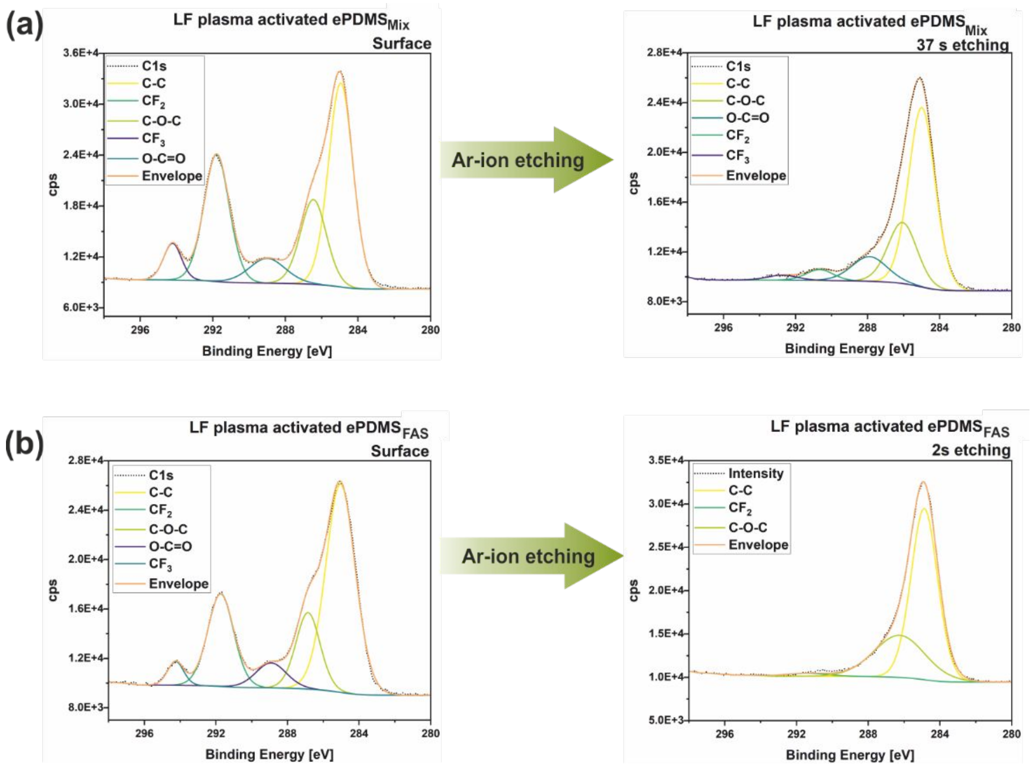

Fig. S9: Carbon scans of (a) **Mix** and (b) **FAS** functionalized **ePDMS^{LF}** samples.

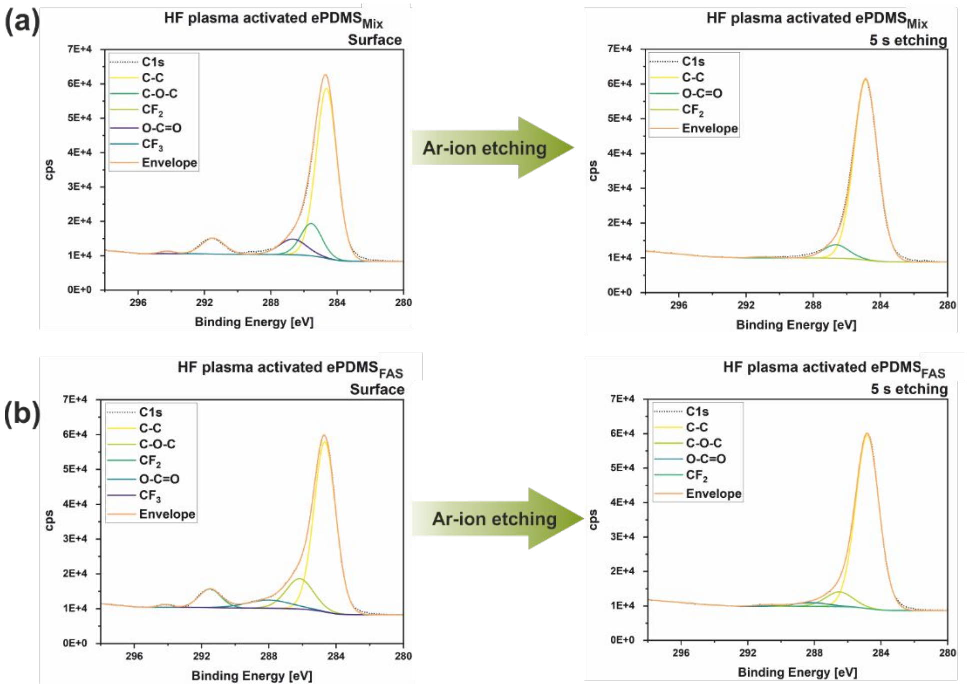

Fig. S10: Carbon scans of (a) **Mix** and (b) **FAS** functionalized **ePDMS^{HF}** samples.

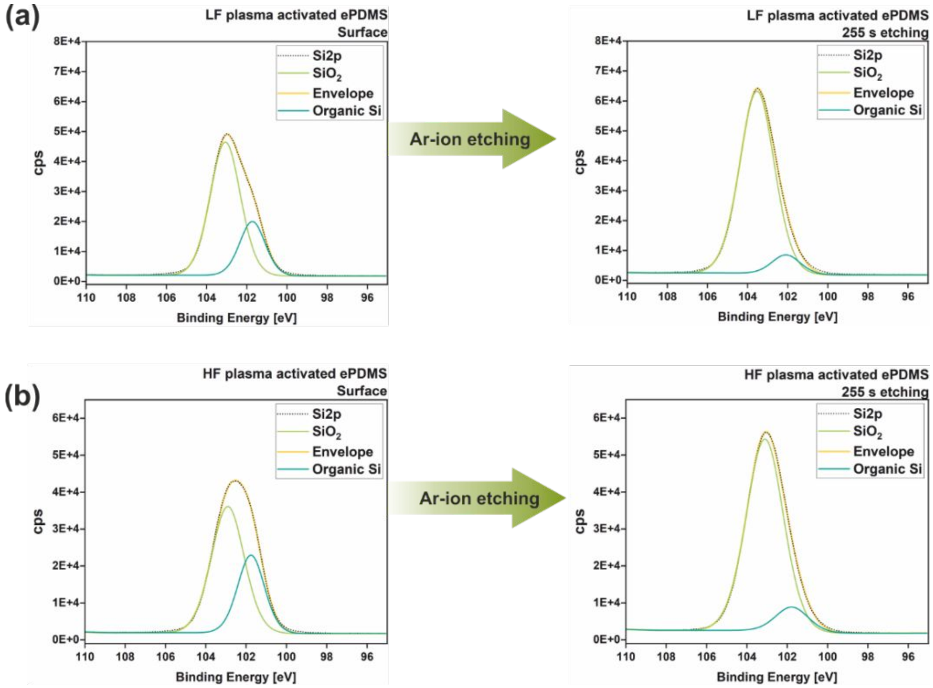

Fig. S11: Deconvolution of Si elemental scans showing organic and inorganic silicon species. a) **ePDMS^{LF}** exhibits a higher ratio of SiO_2 compared to b) **ePDMS^{HF}**. A ratio of organic Si to inorganic Si of 0.34:1 was found for the surface of **ePDMS^{LF}**, which progressed to 0.09:1 during etching. An increased organic Si to inorganic Si ratio of 0.51:1 for the surface of **ePDMS^{HF}** and 0.13:1 after etching confirmed a lower degree of silicon oxidation.

S2.6. Horizontal Push Tests

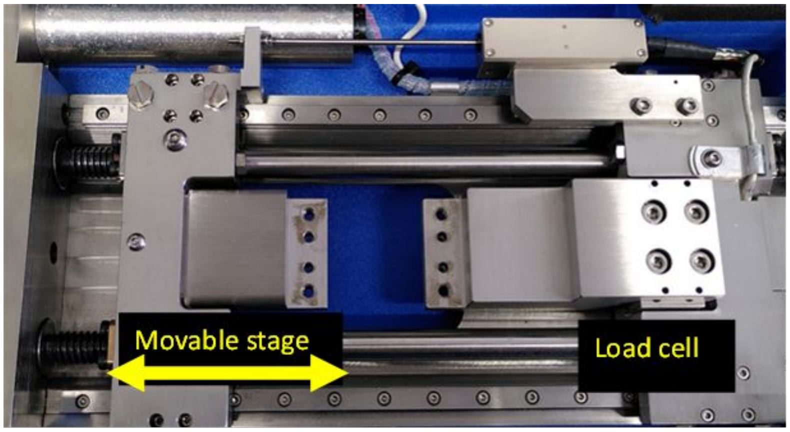

Fig. S12: Deben stage utilized in horizontal push tests.

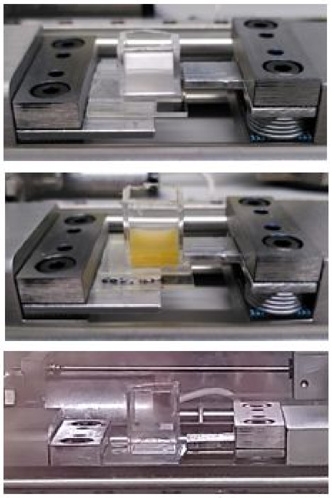

Fig. S13: Photographs of the horizontal push test set up for the tested solid adhesives. The sample is attached to an extendable stage (left) and a probe mounted to the load cell (right).

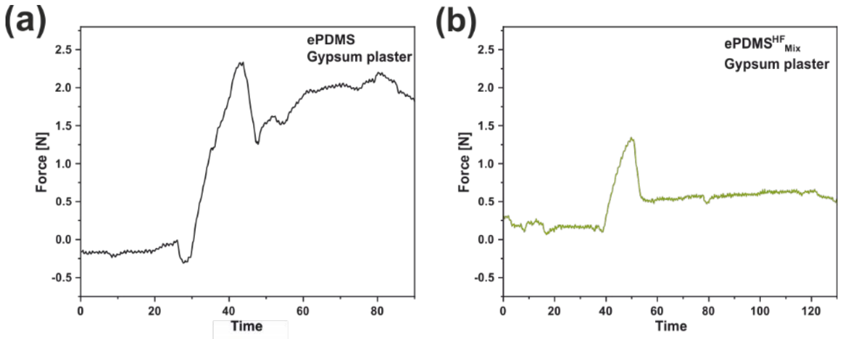

Fig. S14: Force profiles for gypsum plaster in horizontal push tests on (a) **ePDMS** and (b) **ePDMS$^{HF}_{Mix}$**.

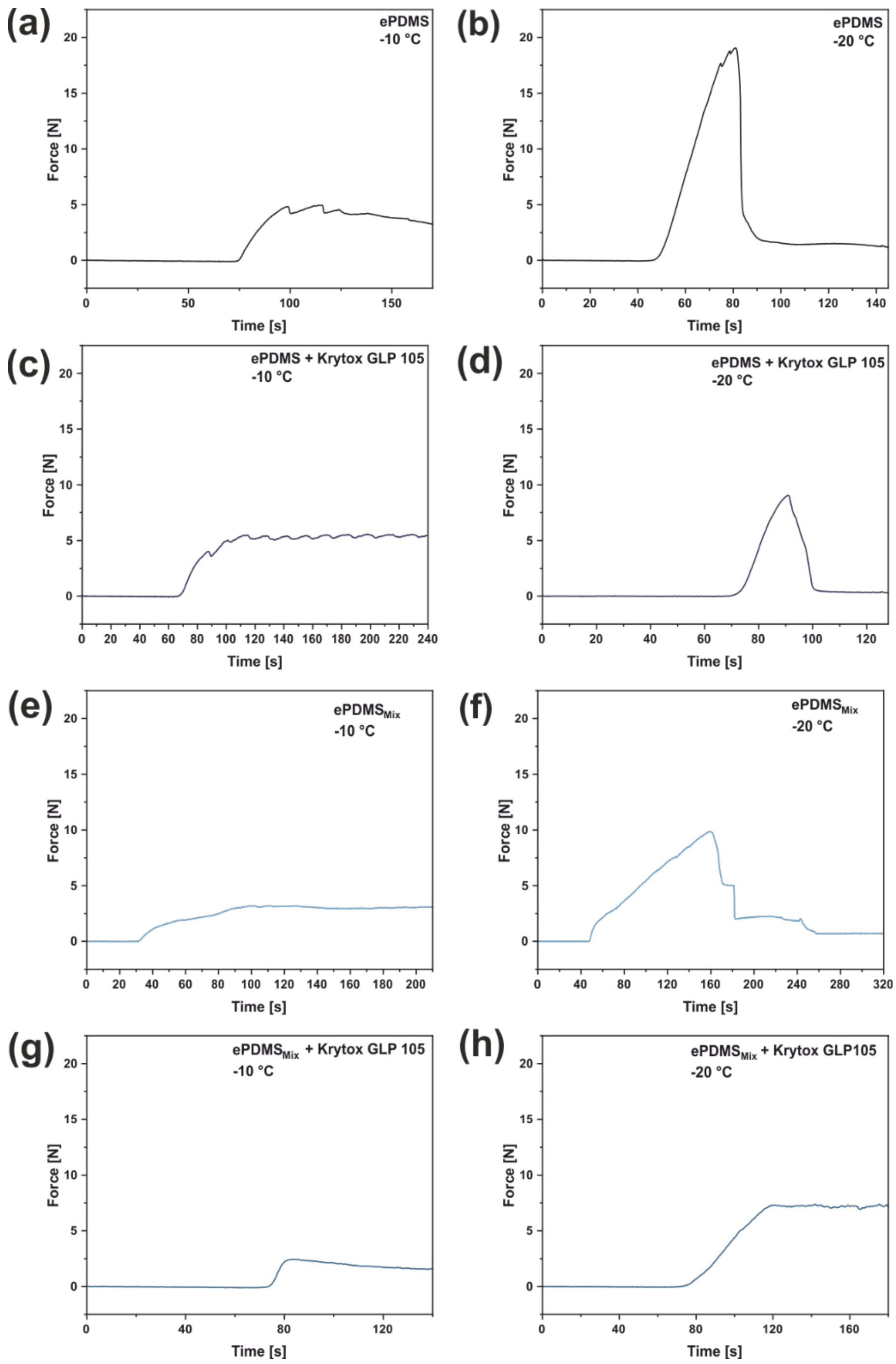

Fig. S15: Force profiles for ice adhesion testing. Force profiles for **ePDMS** tested at (a) -10 °C and (b) -20 °C, **L-ePDMS** at (c) -10 °C and (d) -20 °C, **ePDMS$_{Mix}$** at (e) -10 °C and (f) -20 °C, **L-ePDMS$_{Mix}$** at (g) -10 °C and (h) -20 °C.

Ice adhesion force profiles on **ePDMS** at -10 °C showed high friction across the surface after the interface had been broken and partial stick-slip-like behavior, which impeded the removal of ice. However, at -20 °C a prominent maximum can be observed when the interface was broken and subsequent friction was negligible.

L-ePDMS showed similar to **ePDMS** high friction across the surface and stick-slip-like behavior for some of the samples when ice advanced over the surface, indicating that **SLIPS** formation

was not successful, as they provide a smooth interface, preventing stick-slip-like motion. For -20 °C, force dropped rapidly after the sample-ice interface is broken.

For **ePDMS$_{Mix}$** at -10 °C showed F_{max} ~ friction, similar to the adhesion of beeswax. Similar to plain **ePDMS** at -20 °C **ePDMS$_{Mix}$** force dropped rapidly after the interface is broken.

For **L-ePDMS$_{Mix}$** at -10 °C and -20 °C F_{max} ~ friction behavior was observed.

Reference

(1) Korhonen, J. T.; Huhtamaki, T.; Ikkala, O.; Ras, R. H. Reliable Measurement of the Receding Contact Angle. *Langmuir* **2013**, *29* (12), 3858-3863. DOI: 10.1021/la400009m.
